# Ablation of polyamine catabolic enzymes provokes Purkinje cell damage, neuroinflammation, and severe ataxia

**DOI:** 10.1186/s12974-020-01955-6

**Published:** 2020-10-14

**Authors:** Kamyar Zahedi, Marybeth Brooks, Sharon Barone, Negah Rahmati, Tracy Murray Stewart, Matthew Dunworth, Christina Destefano-Shields, Nupur Dasgupta, Steve Davidson, Diana M. Lindquist, Christine E. Fuller, Roger D. Smith, John L. Cleveland, Robert A. Casero, Manoocher Soleimani

**Affiliations:** 1 Department of Medicine, University of Cincinnati College of Medicine, Cincinnati, OH 45267 USA; 2 Research Services, Veterans Affairs Medical Center, Cincinnati, OH 45220 USA; 3 Department of Medicine, University of New Mexico Health Sciences Center, Albuquerque, NM 87131 USA; 4 Research Services, Veterans Affairs Medical Center, Albuquerque, NM 87108 USA; 5 Department of Internal Medicine, Division of Nephrology, University of New Mexico College of Medicine, 915 Camino de Salud, Bldg. 289, IDTC 3315, Albuquerque, NM 87113 USA; 6 Present Address: Department of Internal Medicine, Division of Nephrology, University of New Mexico College of Medicine, Albuquerque, NM 87131 USA; 7 Department of Neurology, Massachusetts General Hospital and Harvard Medical School, Boston, MA 02129 USA; 8 The Sidney Kimmel Comprehensive Cancer Center, Johns Hopkins University School of Medicine, Baltimore, MD 21231 USA; 9 The Division of Human Genetics, Cincinnati Children’s Hospital Medical Center, Cincinnati, OH 45229 USA; 10 Department of Anesthesiology and Pain Research Center, University of Cincinnati College of Medicine, Cincinnati, OH 45267 USA; 11 Department of Radiology, University of Cincinnati, Cincinnati Children’s Hospital Medical Center, Cincinnati, OH 45229 USA; 12 Upstate Medical University Department of Pathology, Syracuse, NY 13219 USA; 13 Department of Pathology and Laboratory Medicine, University of Cincinnati College of Medicine, Cincinnati, OH 45267 USA; 14 Department of Tumor Biology, Moffitt Cancer Center and Research Institute, Tampa, FL USA; 15 Department of Cancer Biology, The Scripps Research Institute, Jupiter, FL USA

**Keywords:** Polyamine, Polyamine catabolism, Transglutaminase, Protein polyamination, Gliosis, Neuroinflammation, Cerebellum, Purkinje cells, Ataxia

## Abstract

**Background:**

Polyamine catabolism plays a key role in maintaining intracellular polyamine pools, yet its physiological significance is largely unexplored. Here, we report that the disruption of polyamine catabolism leads to severe cerebellar damage and ataxia, demonstrating the fundamental role of polyamine catabolism in the maintenance of cerebellar function and integrity.

**Methods:**

Mice with simultaneous deletion of the two principal polyamine catabolic enzymes, spermine oxidase and spermidine/spermine N^1^-acetyltransferase (*Smox*/*Sat1*-dKO), were generated by the crossbreeding of *Smox*-KO (*Smox*^*−*/*−*^) and *Sat1*-KO (*Sat1*^*−*/*−*^) animals. Development and progression of tissue injury was monitored using imaging, behavioral, and molecular analyses.

**Results:**

*Smox*/*Sat1*-dKO mice are normal at birth, but develop progressive cerebellar damage and ataxia. The cerebellar injury in *Smox*/*Sat1*-dKO mice is associated with Purkinje cell loss and gliosis, leading to neuroinflammation and white matter demyelination during the latter stages of the injury. The onset of tissue damage in *Smox*/*Sat1*-dKO mice is not solely dependent on changes in polyamine levels as cerebellar injury was highly selective. RNA-seq analysis and confirmatory studies revealed clear decreases in the expression of Purkinje cell-associated proteins and significant increases in the expression of transglutaminases and markers of neurodegenerative microgliosis and astrocytosis. Further, the α-Synuclein expression, aggregation, and polyamination levels were significantly increased in the cerebellum of *Smox*/*Sat1*-dKO mice. Finally, there were clear roles of transglutaminase-2 (TGM2) in the cerebellar pathologies manifest in *Smox*/*Sat1*-dKO mice, as pharmacological inhibition of transglutaminases reduced the severity of ataxia and cerebellar injury in *Smox*/*Sat1*-dKO mice.

**Conclusions:**

These results indicate that the disruption of polyamine catabolism, via coordinated alterations in tissue polyamine levels, elevated transglutaminase activity and increased expression, polyamination, and aggregation of α-Synuclein, leads to severe cerebellar damage and ataxia. These studies indicate that polyamine catabolism is necessary to Purkinje cell survival, and for sustaining the functional integrity of the cerebellum.

## Background

The polyamines spermidine and spermine are cationic aliphatic amines that play important roles in the regulation of DNA synthesis and chromatin structure, protein-nucleic acid interactions, translation, and ion channel functions that are important for cell growth and survival [1–3]. The synthesis and export of polyamines in neurons and their uptake by glial cells is tightly regulated [4]. Polyamines are critical in regulating neuronal excitability by modulating the activity of glutamate receptors and inward rectifying potassium (Kir) channels [1, 5, 6]. In addition, polyamines play a critical role in tuning neuronal maturation and plasticity by polyamination of tubulin [7].

**Fig. 1:**
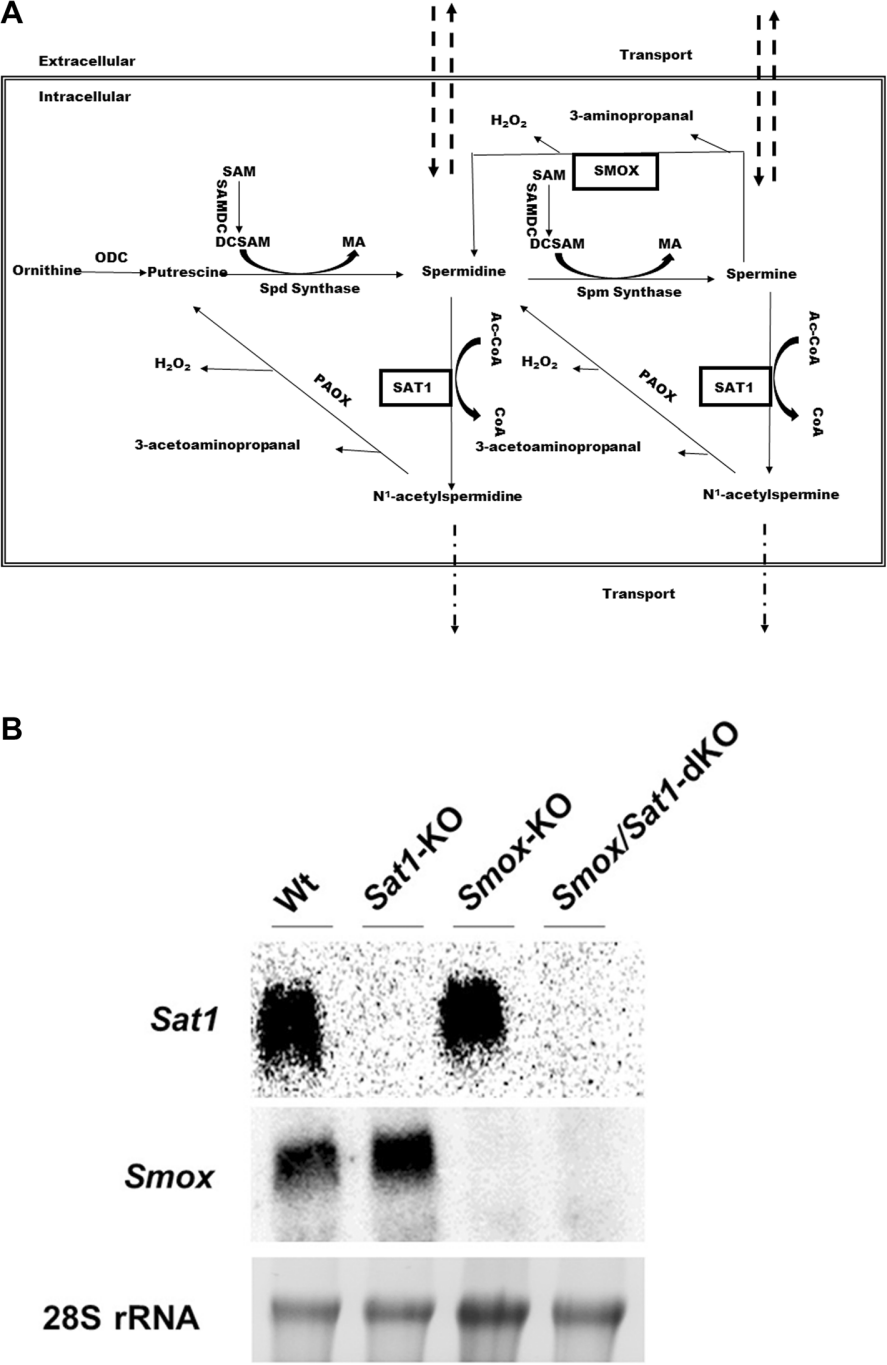
Polyamine pathway and confirmation of loss of *Smox* and *Sat1* expression in *Smox*/*Sat1*-dKO mice. **a** Schematic depiction of the polyamine metabolic pathway, as well as import and export mechanisms. The main pathways of polyamine catabolism that are facilitated by SMOX and SAT1 were disrupted in *Smox*/*Sat1*-dKO mice. **b** The expression of both *Smox* and *Sat1* transcripts were absent in total RNA extracted from the cerebellum of *Smox*/*Sat1*-dKO mice

The control of the import, biosynthesis, catabolism, and export of polyamines in mammalian cells is complex **(**Fig. 1a). Polyamine synthesis is initiated by ornithine decarboxylase (ODC)-mediated decarboxylation of ornithine to form putrescine. Sequential enzymatic addition of aminopropyl groups to putrescine and spermidine leads to the formation of spermidine and spermine, respectively. Polyamines are catabolized via back-conversion reactions that are catalyzed by the spermidine/spermine N^1^-acetyltransferase/N^1^-acetylpolyamine oxidase (SAT1/PAOX) cascade, and by direct oxidation of spermine by spermine oxidase (SMOX). Oxidation of N^1^-acetyl-spermine and N^1^-acetyl-spermidine by PAOX, and of spermine by SMOX generates toxic molecules, including hydrogen peroxide (H_2_O_2_) and reactive aldehydes (e.g., acrolein and 3-aminopropanal) [8].

Polyamine catabolism is enhanced and plays an important role in the mediation of cell and tissue damage in the response of injuries affecting the kidney, brain, and liver [9, 10]. Further, polyamine catabolism has also been implicated in the etiology of a number of epithelial cancers [11, 12]. However, little is known about the physiological roles of polyamine catabolism in development and tissue homeostasis. To address the role of polyamine catabolism under normal physiological conditions, we generated mice with global deletion of both *Sat1* and *Smox* genes. Analyses of these mice establish, for the first time, strikingly selective and unique effects of ablation of polyamine catabolism on the structural and functional integrity of the cerebellum.

## Methods

### Generation of *Smox*/*Sat1*-dKO mice

*Sat1*-KO and *Smox*-KO mice were generated as described previously [10, 13, 14]. Knockout animals were maintained on a C57BL/6 background by back-crossing with C57BL/6 mice every 6 generations. *Smox*/*Sat1*-dKO mice were generated through the following breeding protocols:

1. *Smox*^+/−^/*Sat1*^*+/*−^ x *Smox*^*+/*−^/*Sat1*^*+*/−^
2. *Smox*^−*/*−^/*Sat1*^*+/*−^ x *Smox*^−*/*−^*/Sat1*^*+/*−^

Genotypes of animals were determined by PCR amplification of isolated tail DNA followed by determination of amplified DNA fragment size by agarose gel electrophoresis as previously described. All procedures to maintain and use the mice in these studies were designed using the ARRIVE guidelines and approved by University of Cincinnati’s Institutional Animal Care and Use Committee (IACUC, protocol number 04020901). Throughout the experiments, mice were maintained in a temperature-controlled, AAALAC-certified level 3 facility on a 12-h light–dark cycle. Food and water were provided ad libitum.

### Neurologic scoring system for monitoring mouse ataxia

Animals were examined twice weekly starting at 8 weeks of age to monitor the progression of neurological deficits using the following tests based on previously described protocol [15]: (1) Ledge test; (2) Hindlimb clasping; (3) gait assessment; and (4) observation for kyphosis.

A. Composite Ataxia Scoring

These trials allowed us to assign a Composite Ataxia Score (CAS) for each mouse by adding up the scores from individual tests. Individual test scores were obtained through 10-s testing periods that were repeated 3 times with rest periods in between tests. A higher CAS indicated a more pronounced phenotype. The tests and scoring systems were as follows:

## Ledge test

The ledge test directly measures coordination through placing the mouse directly on the ledge of the home cage and monitoring movement for 10 s. The scoring system is shown below as follows:

0—Mouse will balance itself on the edge of the cage and can gracefully lower itself into the cage.

1—Mouse may lose footing but can lower itself into the cage.

2—Mouse does not use hind legs or cannot lower itself into the cage.

3—Mouse shakes or falls, cannot lower itself into the cage or refuses to move at all.

## Hindlimb clasping

Hindlimb clasping is a marker of disease progression. Mice were picked up at the base of the tail and hind limbs were observed for 10 s. The scoring system is as follows:

- 0—Legs consistently splayed away from the body.
- 1—One leg retracted for over 50% of the time.
- 2—Both hind limbs retracted toward abdomen for over 50% of the time lifted.
- 3—Both hind limbs retracted for the entire time lifted.

## Gait assessment

The gait of the animal is a measure of coordination and muscle function. Mice were placed on a flat surface and were their gait and body posture were assessed for 10-s periods twice with 5 min of rest in between. The scoring system is as follows:

- 0—Normal movement, body weight supported by all limbs, abdomen not touching the surface, and equal load bearing on all limbs.
- 1—Mouse exhibits a limb tremor or feet are slightly pointed away from the body.
- 2—Severe tremor or limp, lowered pelvis, or feet are severely pointed away from the body.
- 3—Difficulty moving forward or dragging abdomen along the surface.

## Observation for kyphosis

Kyphosis is an abnormal curvature of the spine caused by lack of muscle tone. It is used to assess the mouse’s condition based on the Body Condition Scoring chart. Mice were placed on a flat surface and their movements were observed for 10 s. The scoring system is as follows:

- 0—Can straighten spine as it walks. No curvature.
- 1—Mild curvature, but can straighten the spine.
- 2—Unable to straighten its spine. Mild but persistent kyphosis.
- 3—Pronounced kyphosis as it walks or sits.

B. Rotarod test

These studies were performed to examine the neuromuscular coordination. The rotarod test highlights cerebellar or spinal cord injuries or defects. We assessed the latency time required for the mouse to fall from a rod that was rolling at a constant speed. Mice try to remain balanced on the rotating rod. A constant speed for rotarod analysis is used to measure ataxia [16]. These tests quantified the ability of mice to balance. Briefly, after habituation in the test room, motor coordination was measured using a rotarod apparatus (rod rotation was set at a constant speed of 24 rpm). Mice were tested each week on 2 consecutive days with 3 trials per day with 15-min rest intervals between trials. The time that it took for each mouse to fall from the rod (latency to fall) was recorded.

C. Gait analysis

Gait analysis was performed to compare the gait of *Smox*/*Sat1*-dKO and wild-type (Wt) mice. The front and rear paws of each mouse were coated with red and purple non-toxic paint, respectively. The paint was applied with a paintbrush. The animals were then allowed to walk along a 56-cm-long, 10-cm-wide (with 10-cm-high walls) paper-lined open runway into a darkened overturned empty cage. All mice had 3 training runs and one recorded testing per week. The paper runway liner was labeled with the mouse information and was changed between each run. The resulting footprint patterns were measured based upon three metrics, all measured in cm: (1) stride length was measured as the distance between the front and rear paw prints ipsilaterally; (2) sway length was measured as the distance between the right and left front paw prints, perpendicular to the stride measurement; and (3) stance was measured as the distance between the right and left rear paw prints measured diagonally. When the footprints did not overlap, the distance between the centers of the footprints was recorded. The footprints from the starting portion of the runway where the animal was initializing its movement were excluded.

### Magnetic resonance imaging and spectroscopy

Magnetic resonance imaging and spectroscopy (MRI) data were generated using a Bruker BioSpec 7-T system (Bruker BioSpec 70/30, Karlsruhe, Germany) equipped with 400 mT/m actively shielded gradients and Paravision 6.0 software. For MRI studies, animals were anesthetized using 1% to 2% isoflurane delivered by oxygen. Respiration rate was maintained at around 80 breaths per minute. Animals were kept warm with a flow of warm air controlled by a Small Animal Instruments (Small Animal Instruments, Inc., Stony Brook, NY) monitoring system. Mice were centered in a 38-mm ID linear coil (Bruker). Localizers were acquired in all three planes, followed by an ungated T2-weighted 3D RARE sequence (repetition time 1.2 s, effective echo time 74.7 ms, RARE factor 16, 0.15 mm isotropic resolution) for volumetric measurements. A 1.5 × 1.5 × 3 mm voxel was selected in the cerebellum. The voxel was shimmed using FASTMAP to an average line width of 12 Hz. Water-suppressed data were acquired using VAriable Pulse Power and Optimized Relaxation delays water suppression with a 120 Hz bandwidth followed by Point RESolved Spectroscopy with Outer Volume Suppression localization with a TE of 20 ms, TR of 2500 ms, 256 averages, 2048 points, and a spectral width of 3301 Hz. Unsuppressed data were acquired by turning the VAPOR RF pulses off and acquiring 4 averages. Total scan time was about 1 h. Volumes were estimated by drawing regions of interest in ImageJ. The spectra were imported into LCModel for quantitation, using the unsuppressed acquisition for phasing, eddy-current correction, and an internal water reference. A provided simulated basis set containing 16 metabolite signals (alanine, aspartate, creatine, phosphocreatine, GABA, glucose, glutamine, glutamate, glycerophosphocholine, phsophocholine, inositol, lactate, *N*-acetyl aspartate, *N*-acetylaspartyl, scyllo-inositol, and taurine) was used to fit the spectra to obtain peak areas, which are reported as ratios to creatine, the concentration of which was assumed to be constant. Estimates were retained if the Cramer-Rao lower bound reported by LCModel was less than 25%.

### Acquisition and processing of tissue samples

Acquisition of tissue sample for protein and RNA extraction was performed after sacrificing the animals by cervical dislocation followed by decapitation. The brain was extracted from the skull and divided into cerebrum (left and right hemispheres) and cerebellum, which were snap frozen in liquid nitrogen and stored at − 80 °C.

Samples used for microscopic studies were harvested by rendering the animal unconscious using Euthazol (390 mg/ml pentobarbital sodium and 50 mg/ml phenytoin sodium; Virbac AH, Inc. Fort Worth, TX) and perfusing the animal through the left ventricle with 20 ml of 4 °C saline followed by 20 ml of 4 °C 4% paraformaldehyde in phosphate-buffered saline at pH 7.4. The perfusion-fixed brain was harvested, cut into right and left sections along the medial longitudinal fissure of the cerebrum and bisecting the cerebellum, fixed in 4% paraformaldehyde in phosphate-buffered saline pH 7.4 for 18 h, and then stored in 70% ethanol. Paraformaldehyde-fixed, ethanol-preserved samples were then paraffin embedded, cut into 5 μm sections, and used for Hematoxylin and Eosin, Luxol Fast Blue staining, or immunofluorescence microscopy.

### Measurement of polyamine levels

Cellular polyamine content was determined as describe [16]. However, in place of the 10 mM KH_2_PO_4_ buffer suggested in Kabra et al. [16], distilled H_2_O was used. Perchloric acid extracts of cells were dansylated and chromatographs were resolved by reverse phase high-performance liquid chromatography with an increasing acetonitrile/H_2_O gradient.

### RNA extraction.

For RNA extraction, tissue samples were homogenized and total RNA was isolated using Tri-Reagent (MRC, Cincinnati, OH) following the manufacturer's protocol. RNA pellets were dissolved in Formazol and stored at − 80 °C until use.

### RNA-seq and data analysis.

Total RNA isolated from cerebellum of ataxic *Smox/Sat1*-dKO and Wt mice was used for RNA-seq analyses. RNA-seq was performed at the Cincinnati Children’s Hospital Medical Center Genomic Core Facility. Post alignment the Binary Alignment Map files of RNA-seq data were imported and analyzed using Avadis® NGS Version 1.3.0 software. Reads were filtered to remove (1) duplicate reads, (2) non-primary matched reads, and (3) reads with alignment scores of < 95. Quantification was performed on the filtered reads against the mouse RefSeq annotation mm9.

Data normalization was performed with the DESeq package. DESeq via R script was performed on the filtered reads using three functions (estimate size factors, estimate dispersions and negative binomial test). The sequencing depth was estimated by the read count of the gene with the median read count ratio across all genes. The method is based on the negative binomial distribution, which allows for less restrictive variance parameter assumptions than does the Poisson distribution. The false discovery rate was calculated according to the Benjamini and Hochberg algorithm. Fold Change ± 1.5 with an FDR of 0.05 was used as criteria for the selection of the differentially expressed genes (DEG).

The functional classification of DEG was performed by the ingenuity pathway analysis (IPA) tool (www.ingenuity.com). The DEG in the KO compared to WT were imported into the IPA knowledge base v6.3 for functional annotation that summarizes the DEGs associated with top biological functions and canonical pathways. A *p* value cut-off of 0.05 was used to identify significant functions and pathways. Cluster analysis of gene expression profiles. Heat maps were generated from hierarchical cluster analysis of the DEGs identified in the KO compared to the WT samples. Hierarchical clustering was performed by Ward’s method using Euclidean distance metric.

### Northern blot analysis

RNA (25 μg/lane) was size fractionated on a 1.6% agarose gel containing formaldehyde. The RNA was transferred overnight at room temperature to a Nylon Membrane (GE Healthcare). Specific PCR-generated cDNA probes were radio-labeled with 5′-deoxycytidine triphosphate [α-32P] (Perkin Elmer), heat denatured, and used to detect the transcripts of interest (membranes were hybridized overnight at 65 °C). Results were documented using a STORM Phosphoimager utilizing ImageQuant software (GE Healthcare).

### Preparation of protein extracts and western blot analysis

Samples (100–200 mg) were sonicated for 10–15 s in homogenization buffer (25 mM Tris–HCl, pH 7.4) containing Halt protease and phosphatase inhibitors (Thermo Scientific). Tissue homogenates were centrifuged at 23,500 g for 10 min, supernatants were collected, and proteins were measured using the BCA protein assay (Thermo Scientific).

For Western blot analysis, 50 μg of each protein extract was size fractionated under denaturing conditions by gel electrophoresis (16% Tris-Glycine polyacrylamide gel). Size fractionated samples were transferred on to nitrocellulose membrane in buffer containing 20 mM Tris, 192 mM glycine, and 20% methanol (pH 8.3) for 90 min at 25 mV. The blocking reaction was performed for 30 min in Tris-buffered saline (137 mM NaCl and 20 mM Tris, pH 7.4) with 0.1% Tween 20 (TTBS) containing 5% non-fat dry milk. Incubation with primary antibody was in the same buffer overnight at 4 °C. The membrane was then washed in TTBS and incubated with appropriate secondary antibody diluted in TTBS plus 5% dry milk for 1 h at room temperature. The membrane was then washed in TTBS, the blot was developed using peroxidase detection reagents (ECL kit, Invitrogen), and exposed to X-ray film for visualization. For a list of primary and secondary antibodies, refer to Supplemental Table 1.

### Immunofluorescence microscopy

Slides were deparaffinized in xylene and then dehydrated in a series of ethanol dilutions followed by citric acid antigen retrieval. Afterwards, slides were incubated in respective primary antibodies overnight at 4 °C. The following day slides were washed in PBS and incubated in the appropriate secondary antibody (1:200) (Alexa Fluor IgG, Invitrogen, Carlsbad, CA) for 2 h at 25 °C. Slides were washed again in PBS and allowed to air dry. Vectashield Hard Set (Vector Labs, Burlington, CA) was added and cover slips were applied. Slides were examined and immunofluorescene microscopic images of cerebellum were acquired as previously described [17, 18] using a Zeiss Axio Imager.M2 microscope and Zeiss Zen Software (Zeiss, Thornwood, NY). For a list of primary and secondary antibodies, please refer to Supplemental Table 1.

### Fluoro-Jade C staining

Fluoro-jade C (FJC) specifically stains degenerating neurons likely through its interaction with C-Tau, which is a marker of nerve cell injury [19]. Degenerating neurons were identified by FJC staining using Biosensis Ready-to-Dilute (RTD)™ FJC Staining Kit (TR-100-FJ, Biosensis) following the instructions of the manufacturer. Briefly, slides bearing 5-μm-thick tissue sections were immersed in a basic alcohol solution consisting of 1% NaOH in 80% ethanol. They were then rinsed in 70% ethanol, distilled water, and then incubated in 0.06% potassium permanganate solution. Slides were stained with 0.0001% solution of FJC and examined by immunofluorescence microscopy. Degenerating neurons stained with JFC emit green fluorescence light upon excitation with blue laser (488 nm).

### Electron microscopy

Samples used for transmission electron microscopy (TEM) were obtained by rendering the animals unconscious using Euthazol and perfusing the animal through left ventricle with 20 ml of saline followed by 20 ml EM fixative (3% glutaraldehyde in 0.1 M Na cacodylate buffer pH 7.4, Poly Scientific R&D Corp.). Tissues were preserved in EM fixative buffer, washed in 0.1 M Na cacodylate buffer pH 6.8 (Bioworld), and post-fixed in 1% osmium tetroxide (EMS). Osmicated samples were washed in cacodylate buffer, dehydrated through a graded ethanol series, and embedded in LX-112 (Ladd Research Industries). Tissue blocks were sectioned (0.5 to 1-μm) and stained with toluidine blue for light microscopic examination. Blocks were cut with an ultramicrotome (Leica EM UC7) to the thickness of 90 nm and counterstained with uranyl acetate 2% (EMS) and lead citrate. Images were acquired with an 80-kV transmission electron microscope (Hitachi, H-7650, V01.07).

### Immunoprecipitation

For immunoprecipitation, Dynabead Protein-G slurry (Invitrogen) was washed with 200 μl of PBS with Tween-20 (PBS-T), incubated with elution buffer for 10 min, and then washed with PBS-T. Next, 10 μg of mouse anti-α-Synuclein monoclonal antibody (Santa Cruz Biotechnology) was diluted in 200 μl of PBS-T, and added to Dynabead Protein-G slurry. The mixture was subjected to rotation for 10 min at room temperature, washed with PBS-T, and the bound antibody was crosslinked to Dynabead Protein-G following the manufacturers’ protocol. Kidney lysates were pre-cleared by incubation with Dynabead Protein-G slurry. Pre-cleared mouse kidney lysates ( in 100 μl of PBS-T) were then added to Dynabeads-Ab complex. The mixture was subjected to rotation for 10 min at room temperature, and the Dynabeads-Ab-antigen complex was washed 3 times using 200 μl of PBS-T. In the last step, 20 μl of elution buffer was heated for 10 min at 70 °C, and then the eluted protein was subjected to western blot using a rabbit anti-Spermine polyclonal antibody (Novus Biologicals).

### TGM2 activity assay

Cerebellar extracts were prepared by homogenizing the tissue of interest in TBS containing 1% NP40 and Halt protease and phosphatase inhibitor cocktail without EDTA (ThermoFisher) using a Biomasher II homogenizer system (Kimble). Measurement of TGM 2 activity in the cerebellar samples (80 μg of protein/well) was performed using the Novus Biologicals colorimetric TGM2 Assay Kit (Novus Biologicals) following the manufacturer's protocol. The results are presented as μUnits of TGM2 activity/mg of protein. Each unit of TGM2 was defined as “the amount of TGM2 that catalyzes the formation of 1 μmole of hydroxyamate per minute from z-Gln-Gly-OH and hydroxylamine at pH 6.0 at 37 °C.”

### Statistical analysis

All data are expressed as the means ± standard deviation (SD). Statistical differences were assessed using unpaired Student’s two-tailed *t* tests for two groups and a one-way ANOVA for three groups or more. Statistical significance was assumed at *p* < 0.05. Exact *p* values are provided in the figure legends.

## Results

### *Smox*/*Sat1*-dKO mice develop progressive ataxia

The *Smox* and *Sat1* double knockout (*Smox*/*Sat1*-dKO) mice were generated by intercrossing *Smox*-KO [14] and *Sat1*-KO [13] mice. *Smox*/*Sat1*-dKO offspring were born at an expected Mendelian frequency. Loss of *Smox* and *Sat1* expression was confirmed by Northern blot analyses of the cerebellar mRNA, which showed the absence of both transcripts in the cerebellum of *Smox*/*Sat1*-dKO versus Wt mice (Fig. 1b).

**Fig. 2:**
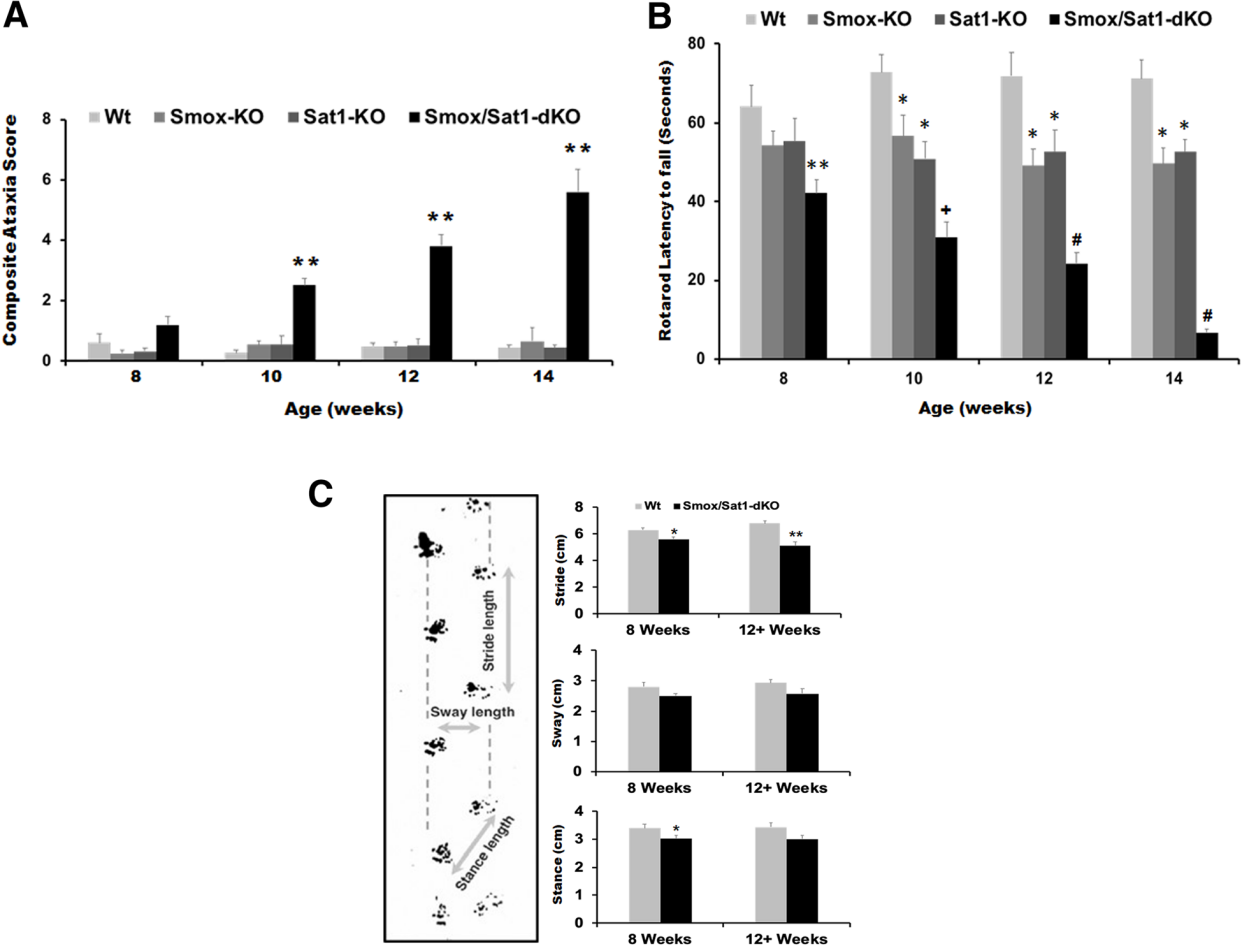
Disruption of polyamine catabolism provokes the development of age-dependent, progressive ataxia. Development of ataxia was monitored by comparing the CAS, rotarod latency to fall and gait of *Smox*/*Sat1*-dKO (*n* = 13) to those of *Smox*-KO (*n* = 9), *Sat1*-KO (*n* = 8), and Wt (*n* = 11) mice. **a** The CAS of *Smox*/*Sat1*-dKO were not significantly different than Wt, *Smox*-KO, and *Sat1*-KO mice at 8 weeks of age. The CAS of *Smox*/*Sat1*-dKO mice were significantly higher than those of Wt, *Smox*-KO, and *Sat1*-KO mice as early as 10 weeks of age (*p* = 0.00245, *p* = 0.00497, and *p* = 0.0000239). The CAS scores continued to increase, remaining significantly higher in *Smox*/*Sat1*-dKO compared to Wt, *Smox*-KO, and *Sat1*-KO in 12 weeks (*p* = 0.00586, *p* = 0.00817, and *p* = 0.00586) and 14 weeks (*p* = 0.00274, *p* = 0.00217, and *p* = 0.00372) of age. **b** Rotorod studies revealed that only the *Smox*/*Sat1*-dKO mice exhibited a progressive and significant reduction in their latency to fall when results at 12 weeks (*p* = 0.00423) and 14 weeks (*p* = 3.37 × 10^−7^) of age were compared to those of 8-week-old mice. The *Smox*/*Sat1*-dKO mice had a significantly shorter latency to fall time compared to Wt, *Smox*-KO, and *Sat1*-KO mice as early as 8 weeks of age (*p* = 0.000846, *p* = 0.00928, and *p* = 0.0297). The results are shown as the means ± SD. For CAS and rotorod results, (*) denotes *p* < 0.05 and (**) denotes *p* < 0.01 for comparison to Wt. For rotorod results, (+) and (#) denote *p* < 0.01 and *p* < 0.001 for comparison to 8-week-old *Smox*/*Sat1*-dKO mice. **c** Gait analyses of Wt versus *Smox*/*Sat1*-dKO mice indicate that there were significant differences in both stance (*p* = 0.0346) and stride (*p* = 0.0334) values on week 8 and significant differences in stride values on week 12 (*p* = 0.000404). For gait analysis results, (*) denotes *p* < 0.05 and (**) denotes *p* < 0.01


Although *Smox*/*Sat1*-dKO mice are viable and normal at birth, they all develop progressive ataxia and exhibit severe deficits in movement as early as 12 weeks of age (Video 1, 8-week-old mouse and video 2, 14+-week-old mouse). Experimental animals were assessed for neurological deficits using a composite phenotype scoring system [15] rotarod and gait analyses. This revealed that *Smox*/*Sat1*-dKO mice developed mild ataxia as early as 8 weeks of age that progressively advanced with age (Fig. 2a). The *Smox*/*Sat1*-dKO mice also showed a significant and progressive reduction in the latency to fall time in rotarod tests at 10, 12, and 14+ weeks of age (Fig. 2b). The results of gait analyses, another metric to assess the movement of animals, revealed significant differences in both stance and stride values on week 8 and significant differences in stride values on week 12 (Fig. 2c). In contrast to *Smox*/*Sat1*-dKO mice, which exhibited progressively worsening movement deficits, *Smox*-KO, *Sat1*-KO, and Wt mice had consistent and stable CAS and Rotorod results (the latency to fall of *Smox*-KO, *Sat1*-KO mice were similar and consistent throughout the study, but shorter than that of their Wt counterparts) (Fig. 2a and b). The *Smox*/*Sat1*-dKO mice died or were euthanized between 16 and 20 weeks of age due to either severe ataxia or a weight loss of greater than 20%, as required by our protocol. The *Smox*-KO, *Sat1*-KO, and Wt did not develop any movement disorders and did not have increased mortality or weight loss that required them to be euthanized.

**Additional file 2: Video S1.** Wt vs *Smox/Sat1*-dKO mouse at 8 weeks of age.


**Additional file 3: Video S2.** Wt vs *Smox/Sat1-*dKO mouse at 14+ weeks of age.

### Ataxia development in *Smox*/*Sat1*-dKO mice is associated with cerebellar damage

**Fig. 3:**
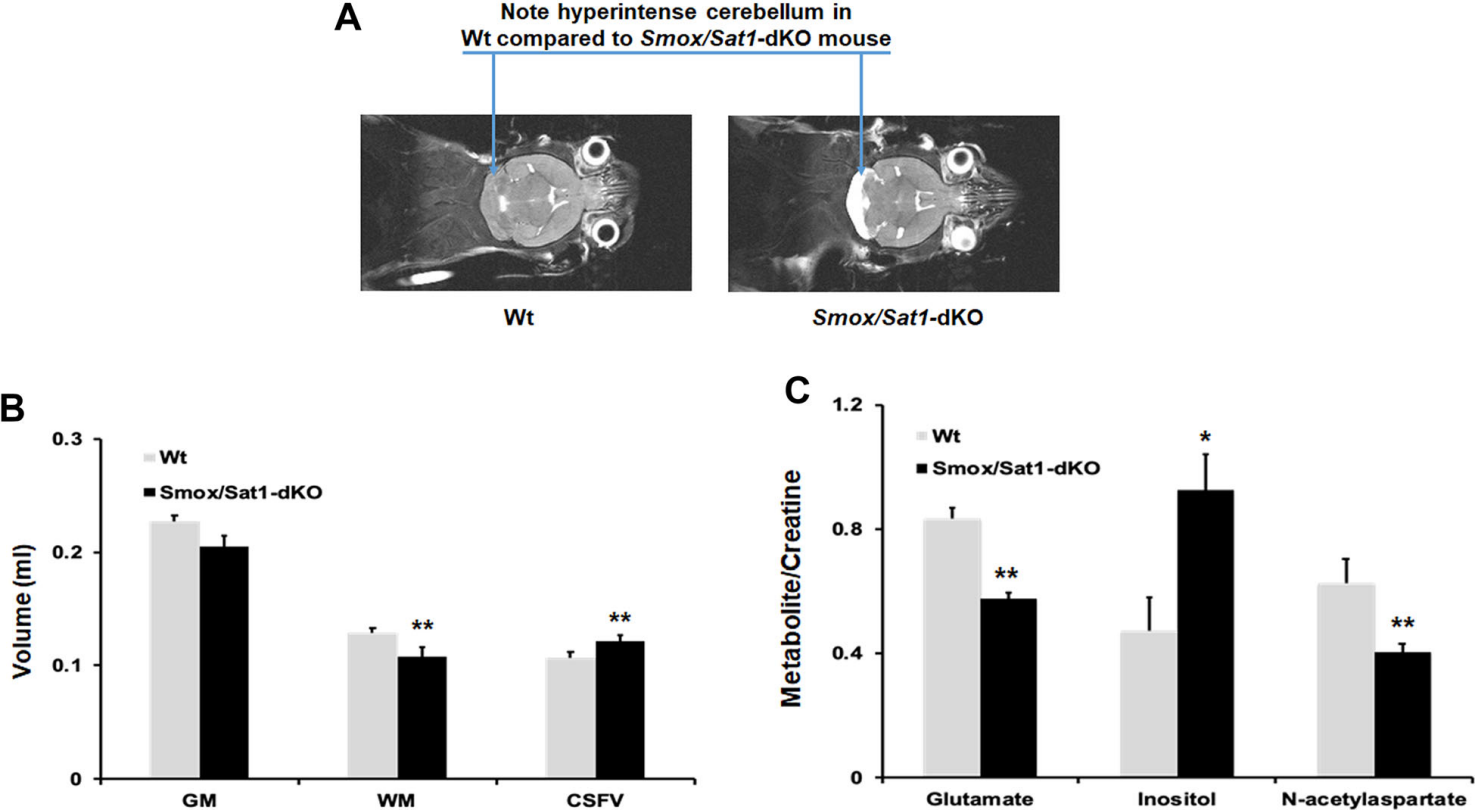
Development of ataxia in *Smox*/*Sat1*-dKO mice is due to selective cerebellar damage. **a** Comparison of the MRI images of Wt (*n* = 6) and *Smox*/*Sat1*-dKO mice (*n* = 4) revealed the latter to have severe cerebellar edema (hyperintense, white colored, region in the cerebellum) without any changes in the cerebrum. **b** Comparison of the volumetric MRI results of Wt (*n* = 6) and *Smox*/*Sat1*-dKO mice (*n* = 4) revealed that the latter have significantly reduced WM volume (*p* = 5.90 × 10^−5^) and elevated CSFV (*p* = 7.35 × 10^−4^). **c** Metabolic MRI also revealed significantly decreased of glutamate (*p* = 0.0036), *N*-acetylaspartate (*p* = 0.0096), and elevated inositol (*p* = 0.013) to creatine ratios. (*) denotes *p* < 0.05 and (**) denotes *p* < 0.01

**Fig. 4:**
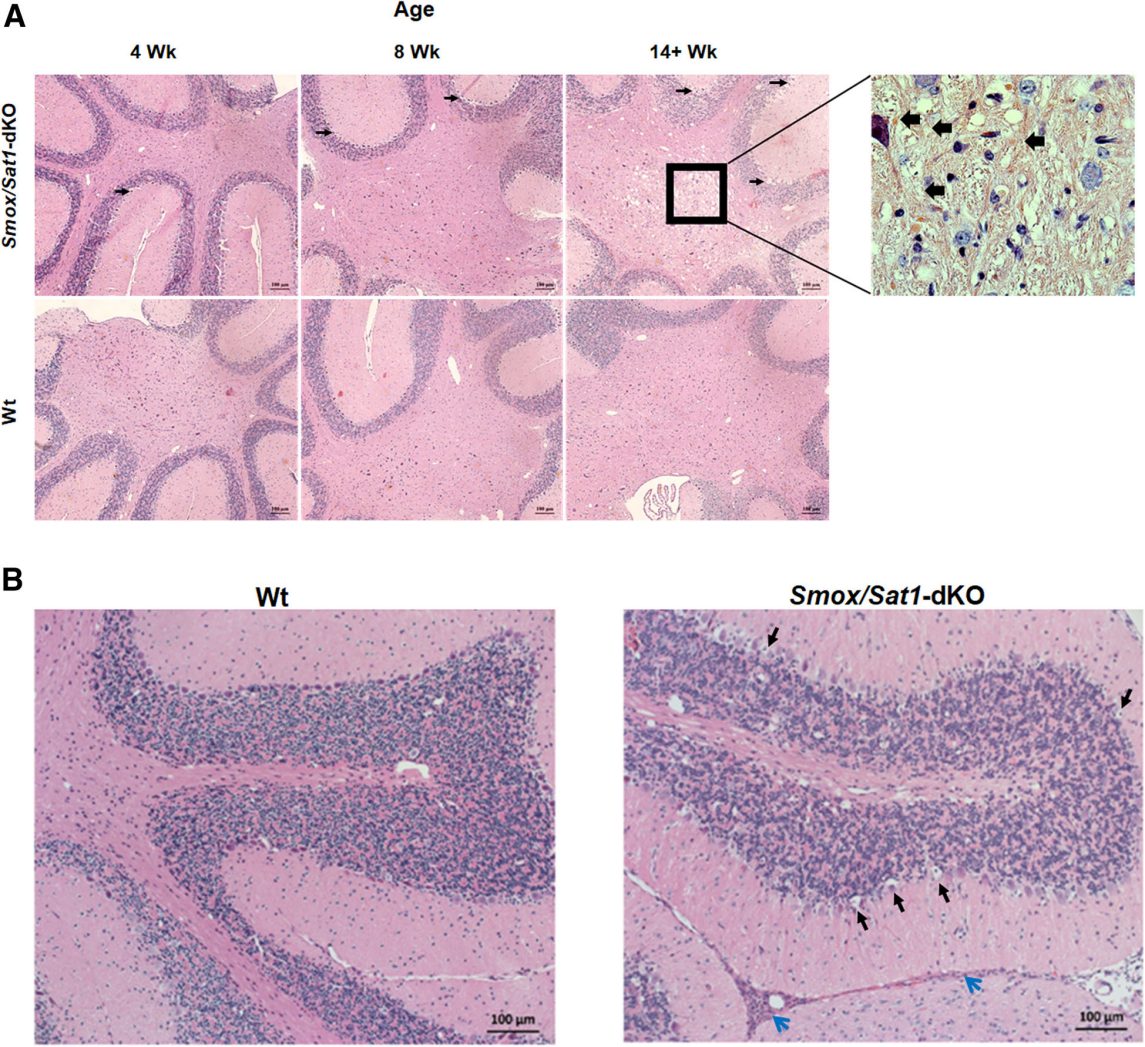
Ataxia in *Smox/Sat1*-dKO mice is associated with early Purkinje cell damage followed by white matter injury and leptomeningeal leukocyte infiltration. **a** Representative H&E stained sections of the cerebellum revealed the presence of Purkinje layer vacuolization (black arrows) and severe demyelination (black box) in the cerebellar white matter of the *Smox*/*Sat1*-dKO mice (large black arrows, *top far right panel*). **b** Examination of comparable cerebellar regions indicates apparent Purkinje cell loss (black arrows) and leptomeningeal leukocyte infiltration (blue arrow) in ataxic mice

To determine the cause of ataxia, mice were subjected to MRI and histological studies. MRI studies demonstrated the following changes in the cerebellum of the *Smox*/*Sat1*-dKO (*n* = 4) compared to age-matched Wt (*n* = 6) mice: (1) cerebellar edema (presence of hyperintense regions) and atrophy (Fig. 3a); (2) significantly reduced cerebellar white matter (WM; Fig. 3a); (3) increased cerebral spinal fluid volume (CSFV; Fig. 3b); (4) diminished glutamate and *N*-acetylaspartate to creatine ratios; and (5) elevated inositol to creatine ratios (Fig. 3c). Notably, MRI did not detect changes in other areas of the brain.

**Fig. 5:**
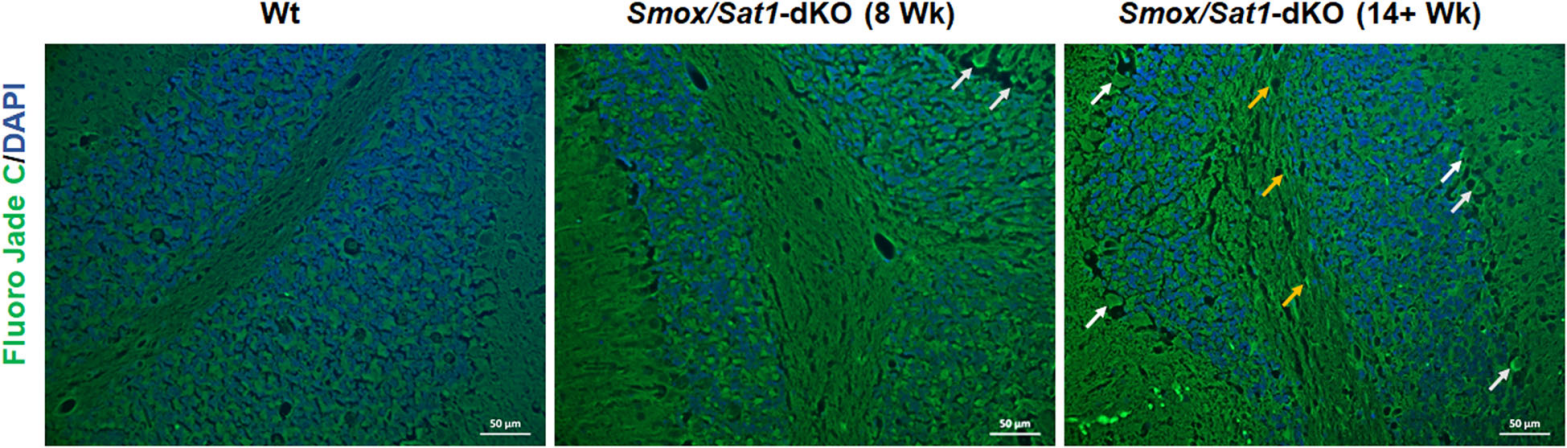
Onset of neurodegenerative changes in cerebellum of *Smox*/*Sat1*-dKO mice. FJC staining reveals the presence of increased neurodegeneration (green fluorescence) as early as 8 weeks (white arrows) in Purkinje cell layer and axonal staining in the white matter (orange arrows) of the cerebellum of *Smox*/*Sat1*-dKO mice

Examination of hematoxylin and eosin (H&E)-stained slides indicated that while the cerebellum was affected in *Smox*/*Sat1*-dKO mice, no histologic abnormalities were detected in the cerebrum, spinal cord, lung, kidney, or liver of the *Smox*/*Sat1*-dKO animals (Supplemental Fig. 1). The cerebellar histology was comparable in the white matter of 4-week and 8-week-old Wt and *Smox*/*Sat1*-dKO (Fig. 4a, *top left* and *middle panels*). However, there was mild vacuolization in the Purkinje cell layer of 4 weeks and 8 weeks old *Smox*/*Sat1*-dKO but not in Wt mice (Fig. 4a, *top left* and *middle panels*). In severely ataxic *Smox*/*Sat1*-dKO mice (14+ weeks old), there was a marked deterioration of the Purkinje cell layer (Fig. 4a, top right panel and Fig. 4b, right panel, black arrows). Also, the cerebellum of severely ataxic mice also showed extensive degeneration of cerebellar white matter (Fig. 4a, *top right panel*), as well as leukocyte infiltration in the leptomeninges (Fig. 4b, *right panel*, blue arrows). The onset of neurodegeneration was further confirmed by FJC staining (Fig. 5a, *middle* and *right panels*). Although it has been proposed that FJC recognizes C-Tau, a marker of injured neurons [19], the mechanistic basis of FJC staining of degenerating neurons has not yet been elucidated. Green fluorescence, which is indicative of neurodegeneration, was observed in Purkinje cells at 8 and 14+ weeks, and in the white matter of 14+-week-old *Smox*/*Sat1*-dKO mice. There was no remarkable FJC staining in the cerebellum of Wt animals (Fig. 5a, *left panel*). The FJC staining of the cerebrum did not reveal any neurodegenerative changes in Wt mice; however, the ataxic mice had multiple FJC-positive neurons, indicating the presence of neurodegenerative changes (14+-week-old *Smox*/*Sat1*-dKO mice had higher number of FJC stained neurons than Wt and 8-week-old *Smox*/*Sat1*-dKO mice; Supplemental Fig. 2).

### Reduced cerebellar and cerebral spermidine and increased spermine levels are manifest in *Smox*/*Sat1-*dKO mice

**Fig. 6:**
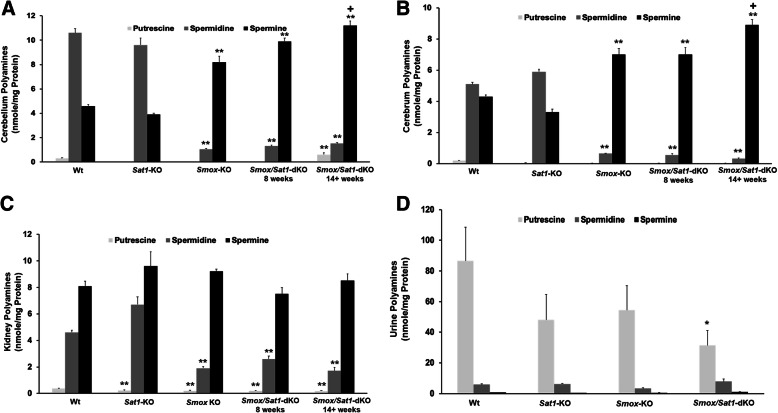
Tissue and urine polyamine levels in Wt, *Sat1*-KO, *Smox*-KO, and *Smox*/*Sat1*-dKO mice. Polyamine contents of the cerebellum, cerebrum and kidney of Wt (*n* = 6), *Sat1*-KO (*n* = 4), *Smox*-KO (*n* = 4) at 14+ weeks of age, *Smox*/*Sat1*-dKO at 8 weeks of age (*n* = 6) and *Smox*/*Sat1*-dKO at 14+ weeks of age (*n* = 7) were measured in duplicate (*t* = 2). **a** The cerebellar spermidine levels decreased significantly in *Smox*-KO (*p* = 5.2 × 10^−16^), 8-week-old *Smox*/*Sat1*-dKO (*p* = 1.82 × 10^−19^) and 14+-week-old *Smox/Sat1*-dKO (*p* = 1.24 × 10^−20^) compared to Wt mice. The levels of spermine were significantly elevated in the cerebellum of *Smox*-KO (*p* = 4.64 × 10^−11^), 8-week-old *Smox*/*Sat1*-dKO (*p* = 4.87 × 10^−20^), and 14+-week-old *Smox*/*Sat1*-dKO (*p* = 5.07 × 10^−21^) compared to Wt mice. Cerebellar polyamine levels of Wt and *Sat1*-KO mice were not significantly different. **b** The cerebral spermidine levels decreased significantly in *Smox*-KO (*p* = 1.04 × 10^−16^), pre-ataxic *Smox*/*Sat1*-dKO (*p* = 4.55 × 10^−23^), and ataxic *Smox*/*Sat1*-dKO (*p* = 1.77 × 10^−26^) compared to Wt mice. The levels of spermine were significantly elevated in the cerebrum of *Smox*-KO (*p* = 2.28 × 10^−9^), pre-ataxic *Smox*/*Sat1*-dKO (*p* = 9.11 × 10^−9^), and ataxic *Smox*/*Sat1*-dKO (*p* = 3.09 × 10^−16^) compared to Wt mice. **c** Spermidine levels in the kidneys of *Smox*-KO pre-ataxic *Smox*/*Sat1*-dKO and ataxic *Smox*/*Sat1*-dKO were similar to Wt mice. The spermidine levels in the kidneys of *Smox*-KO (*p* = 5.78 × 10^−9^), pre-ataxic *Smox*/*Sat1*-dKO (*p* = 2.76 × 10^−6^), and ataxic *Smox*/*Sat1*-dKO (*p* = 4.46 × 10^−9^) mice were significantly lower than Wt mice. **d** The urinary putrescine levels of *Smox*/*Sat1*-dKO were significantly lower than Wt mice (*p* = 0.028), while the urinary levels of other polyamines did not reveal any significant differences. (*) Denotes *p* < 0.05, (**) denotes *p* < 0.0001 compared to Wt, (+) denotes *p* < 0.05 when comparing 14+-week-old *Smox*/*Sat1-dKO* mice to 8-week-old *Smox*/*Sat1*-dKO and *Smox*-KO mice

Despite severe damage being selective to the cerebellum of *Smox*/*Sat1*-dKO mice, there were similar alterations in polyamine levels in both the cerebellum and cerebrum of 8-week-old and 14+-week-old *Smox*/*Sat1*-dKO, and 14+-week-old *Smox*-*KO* mice (Fig. 6a and b). In contrast, polyamine content of the cerebellum and cerebrum of *Smox*/*Sat1*-dKO and *Smox*-KO mice were significantly different than that of 14+-week-old Wt and *Sat1*-*KO* mice (Fig. 6a and b). Specifically, the cerebellar and cerebral spermidine levels decreased significantly in 8- and 14+-week-old *Smox*/*Sat1*-dKO mice as well as in 14+-week-old *Smox*-KO mice compared to 14+-week-old Wt and *Sat1*-KO mice (Fig. 6a and b). Levels of spermine were significantly elevated in *Smox*/*Sat1*-dKO as well as *Smox*-KO mice, compared to Wt mice (Fig. 6a and b). Comparison of polyamine contents revealed a trend toward increased levels of spermine and putrescine in *Smox*/*Sat1*-dKO mice (14+-week-old *Smox*/*Sat1*-dKO > 8-week-old *Smox*/*Sat1*-dKO > *Smox*-KO > Wt for spermine, Fig. 6a and b; and 14+-week-old *Smox*/*Sat1*-dKO > Wt > 8-week-old *Smox*/*Sat1*-dKO > *Smox*-KO for putrescine, Fig. 6a). Samples from cerebrum had very low putrescine levels in all strains except the Wt (Fig. 6b). Kidney spermine levels were comparable in all genotypes, whereas spermidine and putrescine levels were significantly reduced in *Sat1-KO*, *Smox*-KO, 8-week-old *Smox*/*Sat1*-dKO and 14+-week-old *Smox*/*Sat1*-dKO mice compared to Wt mice (Fig. 6c). The examination of urinary polyamine levels did not reveal any differences other than a reduction in the putrescine levels of *Smox*/*Sat1*-dKO versus Wt mice (Fig. 6d).

### Transcriptional programs of Purkinje cell function, neurodegenerative gliosis, and altered protein modification are present in the cerebellum of *Smox*/*Sat1*-dKO mice

RNA-seq analysis of the cerebellar transcriptomes of sex- and age-matched Wt (*n* = 3) and ataxic *Smox*/*Sat1*-dKO (*n* = 3) mice revealed a total of 318 differentially expressed genes (Supplemental Data set 1). Examination of these transcripts (Table 1) indicated that a number of them are associated with (1) Purkinje cell function and movement disorders (e.g., calbindin D28k, *Calb1*; carbonic anhydrase 8, *CA8*; Purkinje cell protein 2, *Pcp2*; solute carrier family 1 member A3, *Slc1A3*, and Transient receptor potential cation channel subfamily C member 3, *Trpc3*); (2) neurodegenerative polarization of microglia (e.g., complement factor 1q, *C1q*; serine peptidase inhibitor clade E member 1, *Serpine1*, and pentraxin 3, *Ptx3*) and astrocytes (e.g., complement factor 3, *C3*; complement factor 4, *C4*; Serine peptidase inhibitor clade A member 3N, *Serpina3n* and glial fibrillary acidic protein, *Gfap*); and (3) protein modification associated with neurodegenerative conditions (e.g., transglutaminases 1 and 2; *Tgm1* and *Tgm2*). In addition to involvement in protein modification, neurodegeneration, gliosis, movement disorders, and immune and inflammatory responses, the differentially regulated transcripts are implicated in a number of other pathways (Supplemental Data sets 2 and 3, and Supplement Fig. 3).

**Table 1:** Lists of transcripts on interest that are differentially regulated in *Smox*/*Sat1*-*dKO* compared to Wt mice

Purkinje function and movement	Neurodegenerative polarization of microglia	Neurodegenerative polarization of astrocytes	Protein modification associated with neurodegeneration
Calb1	C1q	C3	Tgm1
Ca8	Serpine1	C4	Tgm2
Pcp2	Ptx3	Serpina3n	
Slc1a3		Gfap	
Trpc3			

**Fig. 7:**
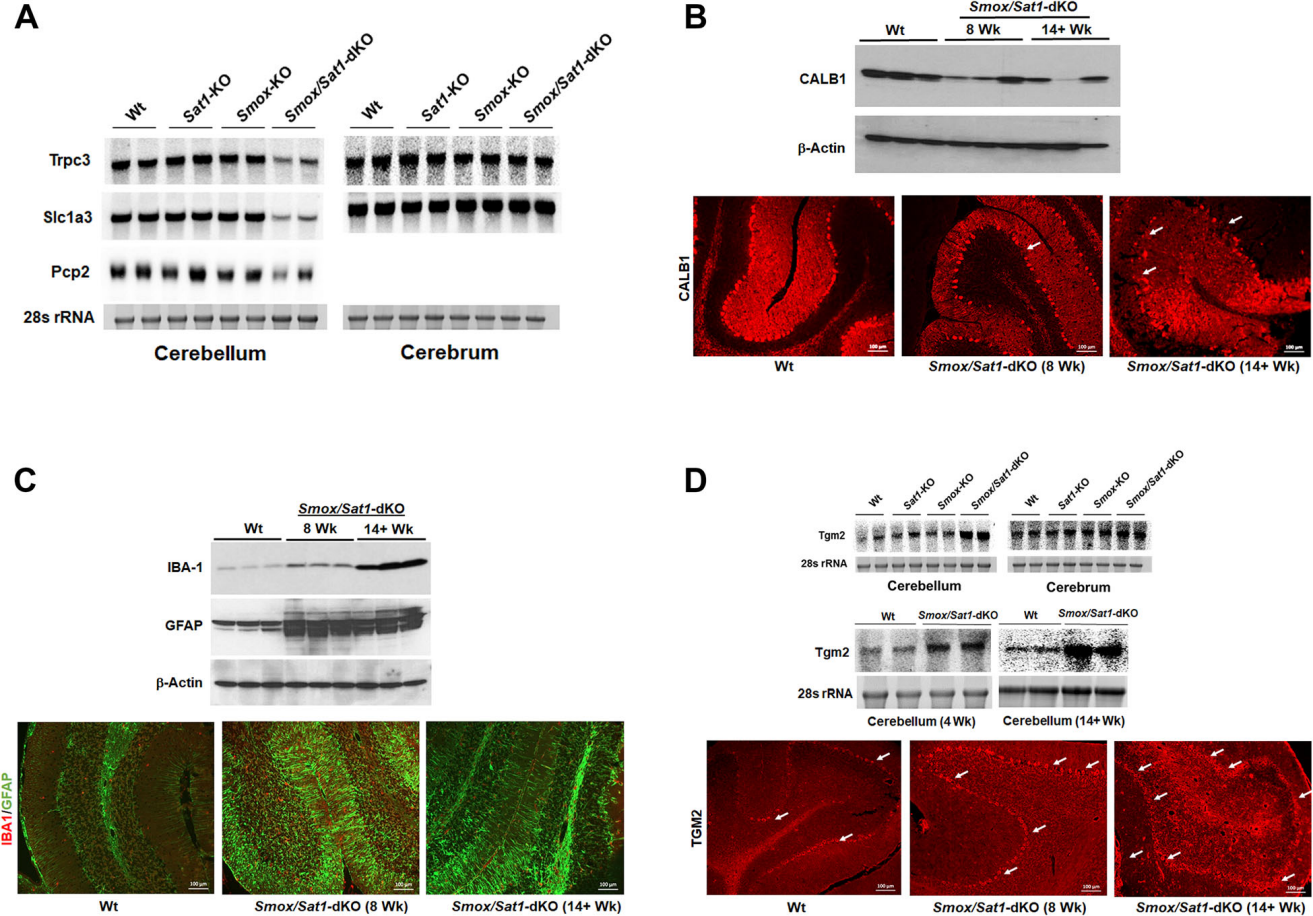
Altered expression of genes associated with Purkinje cell function and neurological disorders are manifest in *Smox*/*Sat1*-dKO mice. The changes in the expression of mRNAs and proteins of interest identified by RNA-seq were confirmed by Northern blot, Western blot, or immunofluorescence microscopic analyses. **a** Northern blot analysis of selected transcripts known to be associated with Purkinje cell function and movement disorders in RNA samples from the cerebellum and cerebrum of Wt, *Sat1*-KO, *Smox*-KO, and *Smox*/*Sat1*-dKO mice revealed that while their levels remained unchanged in the cerebrum their expression was reduced in the cerebellum of *Smox*/*Sat1*-dKO mice. **b** Western blot and immunofluorescence microscopy studies indicate that the expression of CALB1, a Purkinje cell marker, decreased in the cerebellum of *Smox*/*Sat1*-dKO versus Wt mice (white arrows point out the areas of Purkinje cell drop out). **c** Western blot analyses indicate that the expression of IBA1, a microglia marker, and GFAP, an indicator of astrocytosis, increased in the cerebellum of *Smox*/*Sat1*-dKO mice as early as 8 weeks of age. Immunofluorescence microscopy studies indicate that the expression of IBA1 (red) and GFAP (green) increases in the cerebellum of *Smox*/*Sat1*-dKO mice as early as 8 weeks of age. The image of *Smox*/*Sat1*-dKO cerebellum at 14+ weeks of age was taken at a reduced exposure to mitigate the loss of definition caused by intensity of the fluorescence signal. **d** Northern blot analysis of *Tgm2* and *Tgm1* mRNA in the cerebellum and cerebrum of Wt, *Sat1*-KO, *Smox*-KO, and *Smox*/*Sat1*-dKO mice (*top panel*) indicates that the expression of both transcripts is elevated in the cerebellum of *Smox/Sat1*-dKO mice only. Also, it should be noted that the mRNA levels of *Tgm1* are significantly lower than *Tgm2* mRNA levels. Comparison of age-matched Wt and *Smox*/*Sat1*-dKO mice reveals that increased expression of *Tgm2* is apparent as early as 4 weeks of age in *Smox*/*Sat1*-dKO mice (*middle panel*). Immunofluorescence microscopic images of comparable cerebellar regions examining the expression of TGM2 in the cerebellum of Wt, 4 weeks, 8 weeks, and 14+-week-old *Smox*/*Sat1*-dKO mice (white arrows point to TGM2 positive cells). Northern blots contained 20 μg of RNA/lane and Western blots contained 50 μg of protein/lane. All analyses are representative of three independent sets of experiments

RNA-seq results for genes of interest were confirmed by Northern, Western, or immunofluorescence microscopic analyses. The mRNA levels for Purkinje cell-associated transcripts such as *Pcp2*, *Slc1a3*, a glutamate transporter critical for Purkinje cell function [20, 21], *Trpc3* (a cation channel important for regulation of calcium influx and electrophysiological properties of Purkinje cells) [22, 23], were significantly downregulated in ataxic *Smox*/*Sat1*-dKO mice (Fig. 7a), confirming the histological observation that loss of polyamine catabolism damages the Purkinje cells. The expression of *Slc1a3* and *Trpc3* transcripts remained unchanged in the cerebrum of ataxic *Smox*/*Sat1*-dKO mice, whereas *Pcp2* mRNA was not detected in the cerebral RNA samples (Fig. 7a). Selective damage to Purkinje cells was also confirmed by Western blot analysis of CALB1 expression, which was reduced in the cerebellar extracts of 8-week-old and 14+-week-old *Smox*/*Sat1*-dKO versus Wt mice (Fig. 7b, *top panel*). Reductions in CALB1 levels and damage to Purkinje cells was further confirmed by immunofluorescence microscopic examination of Wt, 8-week-old and 14+-week-old *Smox*/*Sat1*-dKO mice (Fig. 7b, *bottom panels*). Our results revealed a marked decrease in CALB1 staining and dropout of Purkinje cells in 8-week-old *Smox*/*Sat1*-dKO mice (Fig. 7b, *bottom middle panel*, white arrow point to the area of Purkinje cell dropout). There was a marked disorganization in the Purkinje cell layer as well as a reduction in the CALB1 staining and the number and of Purkinje cells and an increase in axonal spheroids (Fig. 7b, *bottom right panel*, white arrows point to the areas of Purkinje cell dropout), in the cerebellum of 14+-week-old *Smox*/*Sat1*-dKO mice. The expression of CALB1 was not affected in the cerebrum of *Smox*/*Sat1*-dKO mice (Supplemental Fig. 4).

The onset of microgliosis and astrocytosis in *Smox*/*Sat1*-dKO mice was confirmed by additional immunoblot and immunofluorescence analyses. First, cerebellar expression of ionized calcium-binding adapter molecule 1 (IBA1) and GFAP were elevated in both pre-ataxic and ataxic *Smox*/*Sat1*-dKO versus Wt mice (Fig. 7c, *top panel*). The onset of gliosis in the cerebellum of *Smox*/*Sat1*-dKO mice was further confirmed by immunofluorescent double-labeling for GFAP and IBA1. Specifically, the number of microglia that expressed IBA1 was increased, as was the expression of GFAP in the cerebellum of *Smox*/*Sat1*-dKO mice as early as 8 weeks of age, and this correlated with the severity of ataxia (Fig. 7c, *bottom panels*).

The expression of transcripts coding for transglutaminases 1 (Tgm1) and 2 (Tgm2) enzymes that modify a number of proteins associated with neurodegenerative conditions [24–29] were elevated in *Smox*/*Sat1*-dKO mice (Table 1 and Supplemental Data-dataset 1). Northern blot analyses (Fig. 7d) further confirmed that the expression levels of *Tgm2* are significantly more robust than those of *Tgm1*. In contrast, the expression of neither *Tgm1* or *Tgm2* mRNAs were altered in the cerebrum of *Smox/Sat1*-dKO mice, or in the cerebellum and cerebrum of *Smox-*KO, *Sat1*-KO, or Wt mice (Fig. 7d, *top panel* and Supplemental Fig. 5). The expression of *Tgm2* mRNA increased while *Tgm1* mRNA levels were not detectable in the cerebellum of *Smox*/*Sat1*-dKO mice at 4 weeks of age (Fig. 7d, *middle panel*). The expression of mRNA transcripts for both *Tgm1* and *Tgm 2* were elevated in 14+-week-old mice (Fig. 7d, *middle panel*). Again, the expression of *Tgm2* was significantly higher than that of *Tgm1* (Fig. 7d, *middle panel*). Finally, immunofluorescence microscopic analyses revealed that TGM2 expression was elevated in the Purkinje cells of 8-week-old *Smox*/*Sat1*-dKO mice and became more widespread (e.g., Purkinje cells, cerebellar WM and leptomeninges) in severely ataxic (14+ weeks old) *Smox*/*Sat1*-dKO mice (Fig. 7d, *bottom panel*, white arrows denote TGM2 positive cells).

### Increased cerebellar TGM2 levels and enhanced protein polyamination are a hallmark of *Smox*/*Sat1*-dKO mice

**Fig. 8:**
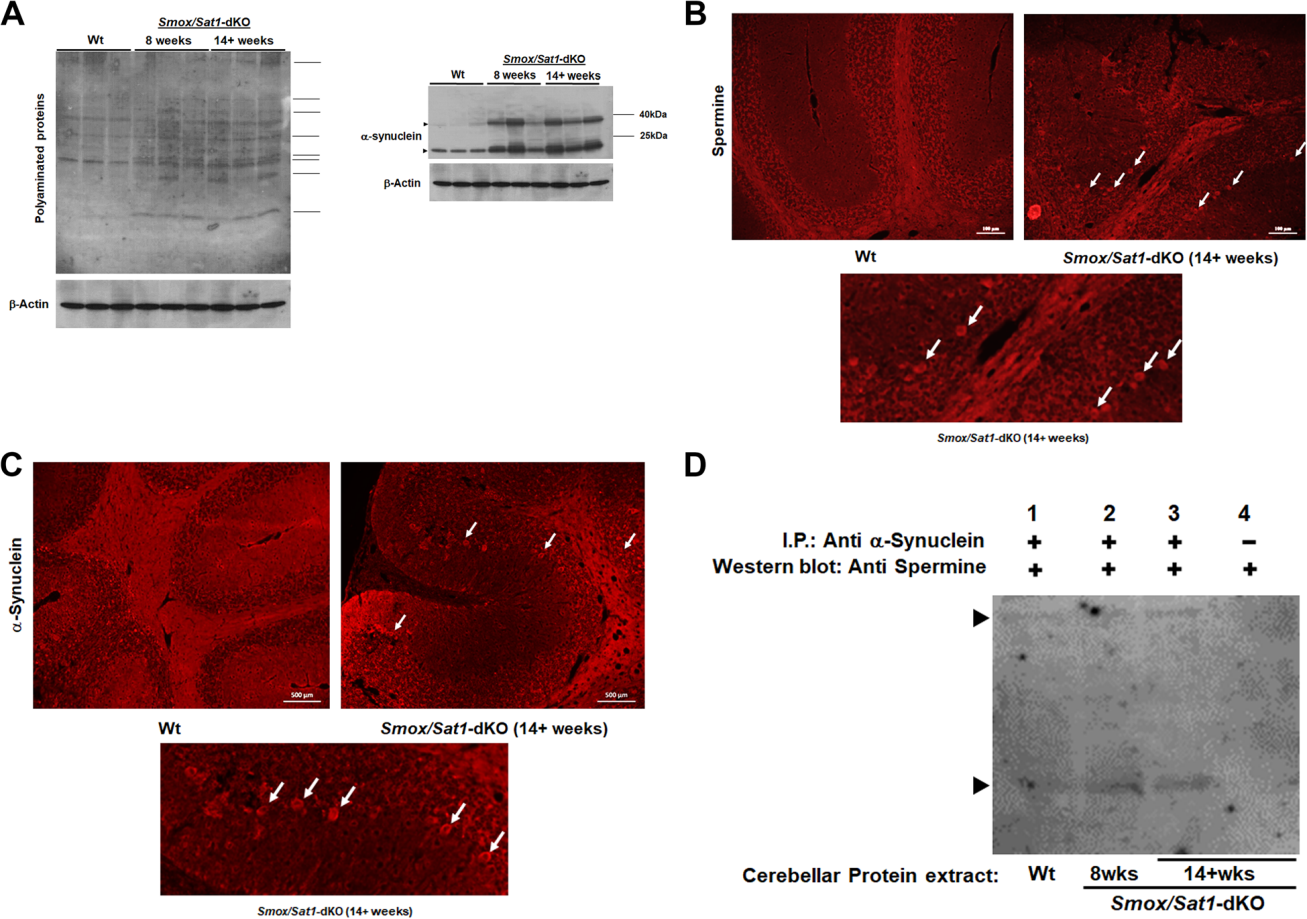
Increased polyaminated proteins and α-Synuclein expression, polymerization and polyamination are a hallmark of *Smox*/*Sat1*-dKO mice. **a** Comparison of protein polyamination and α-Synuclein expression in the cerebellar and cerebral protein extracts of Wt, 8 weeks old (ataxic) and 14+-week-old (severely ataxic) *Smox*/*Sat1*-dKO mice. The lines on the right side of the left panel indicate similar size protein bands that were identified to be differentially polyaminated. **b** Representative immunofluorescence microscopic images of spermine in the cerebellum of ataxic *Smox/Sat1*-dKO versus Wt animals indicates the presence of increased staining in cells of the Purkinje layer. The lower panel shows a higher magnification view of upper left panel. **c** Immunofluorescence microscopy studies examining the expression of α-Synuclein in comparable cerebellar sections of Wt and ataxic *Smox*/*Sat1*-dKO animals indicates the presence of increased staining in cells of the Purkinje layer. The lower panel shows a higher magnification view of upper left panel. **d** α-Synuclein was immunoprecipitated from cerebellar protein extracts of Wt, 8, and 14+-week-old *Smox*/*Sat1*-dKO mice, size fractionated and subjected to Western blot analysis using anti-spermine antibodies. The arrows indicate the bands in anti α-Synuclein antibody immunoprecipitates that reacted with anti-spermine antibody. All results are representatives of at least 3 independent experiments. Northern blots contained 25 μg/lane of RNA and western blots contained 50 μg/lane of protein

Given the role of TGMs in neurodegenerative diseases and their ability to polyaminate proteins [30–32], we assessed if there were alterations in protein polyamination in the cerebellum of *Smox*/*Sat1*-dKO mice. Notably, the polyamination of a number of cerebellar proteins was elevated in 8-week-old and 14+-week-old *Smox*/*Sat1*-dKO compared to Wt mice (Fig. 8a, *right panel*). In contrast, levels of polyaminated proteins were not significantly different in the cerebrum of Wt and *Smox*/*Sat1*-dKO mice (Supplemental Fig. 6). Further, immunofluorescence microscopic analysis of the cerebellum of Wt, 8-week-old, and 14+-week-old *Smox*/*Sat1*-dKO mice using anti-spermine antibodies revealed increased levels in the Purkinje cells of *Smox*/*Sat1*-dKO mice (Fig. 8b, *left panel and bottom panel*).

Notably, the expression and aggregation of α-Synuclein, a pre-synaptic protein often associated with neurodegeneration [33], is augmented in the cerebellum of pre-ataxic and ataxic *Smox*/*Sat1*-dKO mice (Fig. 8a, *left panel*). The α-Synuclein levels were also elevated in the cerebrum of *Smox*/*Sat1*-dKO at 8 (mild ataxia) and 14+ (severe ataxia) weeks of age (Supplemental Fig. 6), but their expression levels were lower in comparison to those found in the cerebellum of the same animals (Fig. 8a). Finally, immunofluorescence microscopy studies also revealed increased expression of α-Synuclein in the Purkinje cells of ataxic *Smox*/*Sat1*-dKO mice (Fig. 8c, *left panel and bottom panel*).

The *C*-terminus of α-Synuclein regulates its oligomerization and also contains a TGM2 target sequence that can be polyaminated [34, 35]. Polyamines are known to enhance the oligomerization of α-Synuclein [36–38]. Immunofluorescence microscopic studies revealed specific co-localization of spermine and α-Synuclein in Purkinje cells of the cerebellum of *Smox*/*Sat1*-dKO mice (Fig. 8b and c). Since TGM2 expression, like that of α-Synuclein, is localized to the Purkinje cells of *Smox*/*Sat1*-dKO mice, we assessed the polyamination status of α-Synuclein by immunoprecipitation/Western blot analysis. Cerebellar extracts subjected to immunoprecipitation with anti-α-synuclein antibodies, size fractionated, and subjected to Western blot analysis using anti-spermine antibodies for detection of polyaminated α-synuclein demonstrated that increased polyamination of monomeric and polymeric α-synuclein is manifested in the cerebellum of ataxic *Smox*/*Sat1*-dKO mice (Fig. 8d, *arrow heads indicate proteins that were immunoprecipitated using anti-*α*-synuclein antibody and recognized by the anti-spermine antibody*).

### Ataxia in *Smox/Sat1*-dKO is associated with white matter demyelination and lymphocyte infiltration

**Fig. 9:**
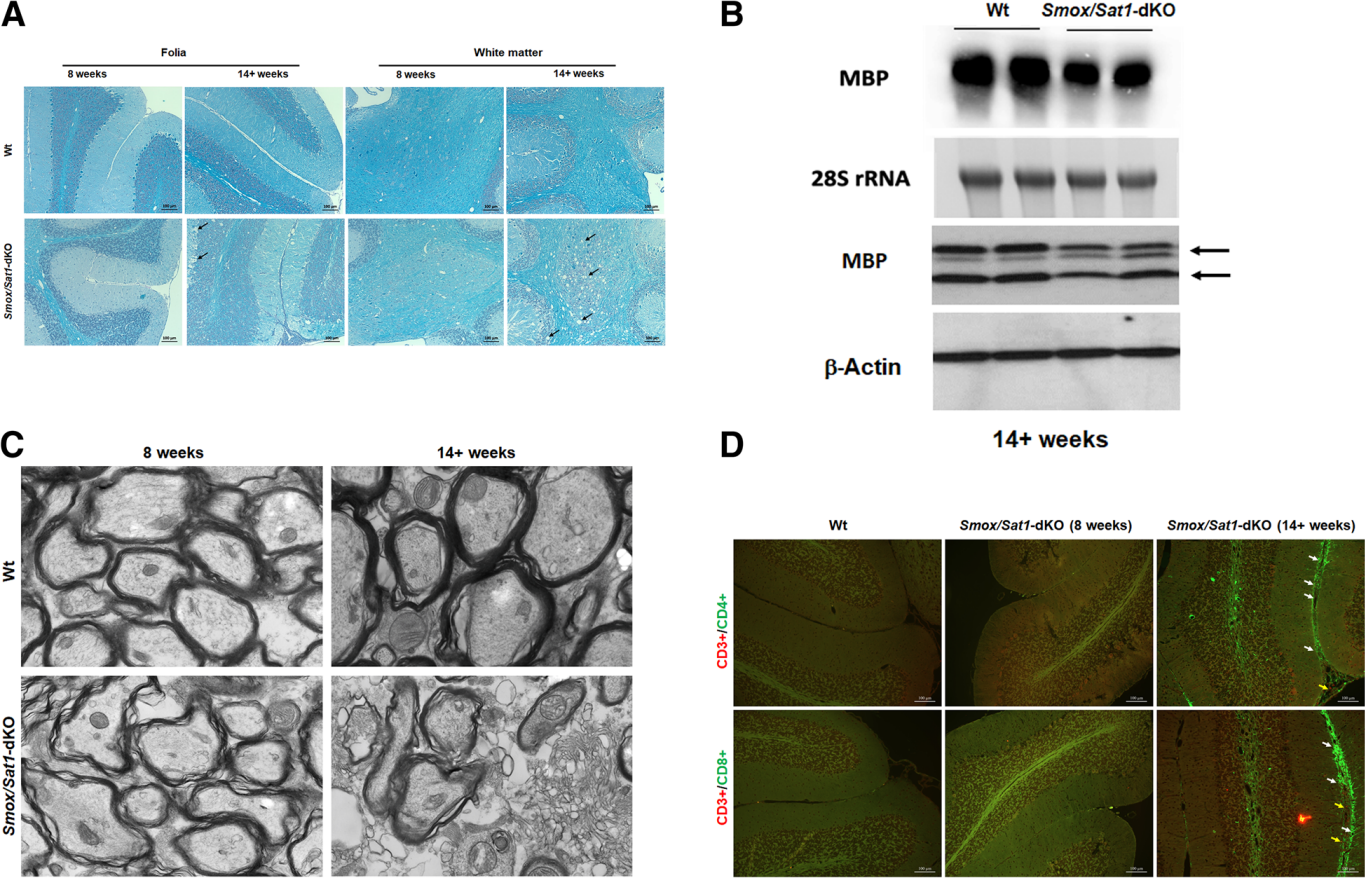
*Smox*/*Sat1*-dKO mice develop severe demyelination and neuronal injury. **a** LFB staining reveals the presence of demyelinating injury in the deep cerebellar nuclei of *Smox*/*Sat1*-dKO mice at 8 weeks of age and severe white matter demyelination and leptomeningeal leukocytic infiltration in the cerebellum of 14+-week-old *Smox*/*Sat1*-dKO mice. **b** Northern and western blot analyses of cerebellar RNA (top two panels) and protein extracts (bottom two panels) revealed a decrease in the expression of MBP mRNA (top panel) and protein (third panel from the top) levels in the cerebellum of *Smox*/*Sat1*-dKO mice. Northern blots contained 25 μg/lane of RNA and western blots contained 50 μg/lane of protein. Results are representative of 3 independent experiments. **c** Transmission electron microscopic analysis of cerebellar myelin sheaths revealed the onset of progressive age-dependent damage (increased translucency and disorganization of the myelin lamellae) in *Smox*/*Sat1*-dKO mice. Results are representative of TEM images obtained from multiple samples; *n* = 3/genotype/time point. **d** Severely ataxic mice develop profuse leptomeningeal lymphocyte infiltrates rich in CD4^+^ and CD8^+^ cells

Cerebellar sections stained with Luxol Fast Blue (LFB) showed mild demyelination in the deep cerebellar nuclei in 8-week-old *Smox*/*Sat1*-dKO mice (Fig. 9a). The extent and severity of demyelination was profoundly increased and spread to the white matter of the folia in 14+-week-old animals. These results were confirmed by immunoblot analyses, which showed a reduction in myelin basic protein (MBP) levels in the cerebellum of *Smox*/*Sat1*-dKO mice versus Wt mice (Fig. 9b), and by TEM, which showed age-dependent progressive disorganization and increased interlamellar electron lucency in the myelin sheath of the cerebellum of *Smox*/*Sat1*-dKO mice (Fig. 9c). Finally, the development of severe ataxia in *Smox*/*Sat1*-dKO mice was accompanied by accumulation of CD3^+^, CD4^+^, and CD8^+^ lymphocytes in the leptomeninges (Fig. 9d).

### TGM2 contributes to cerebellar injury in *Smox*/*Sat1*-dKO mice

**Fig. 10:**
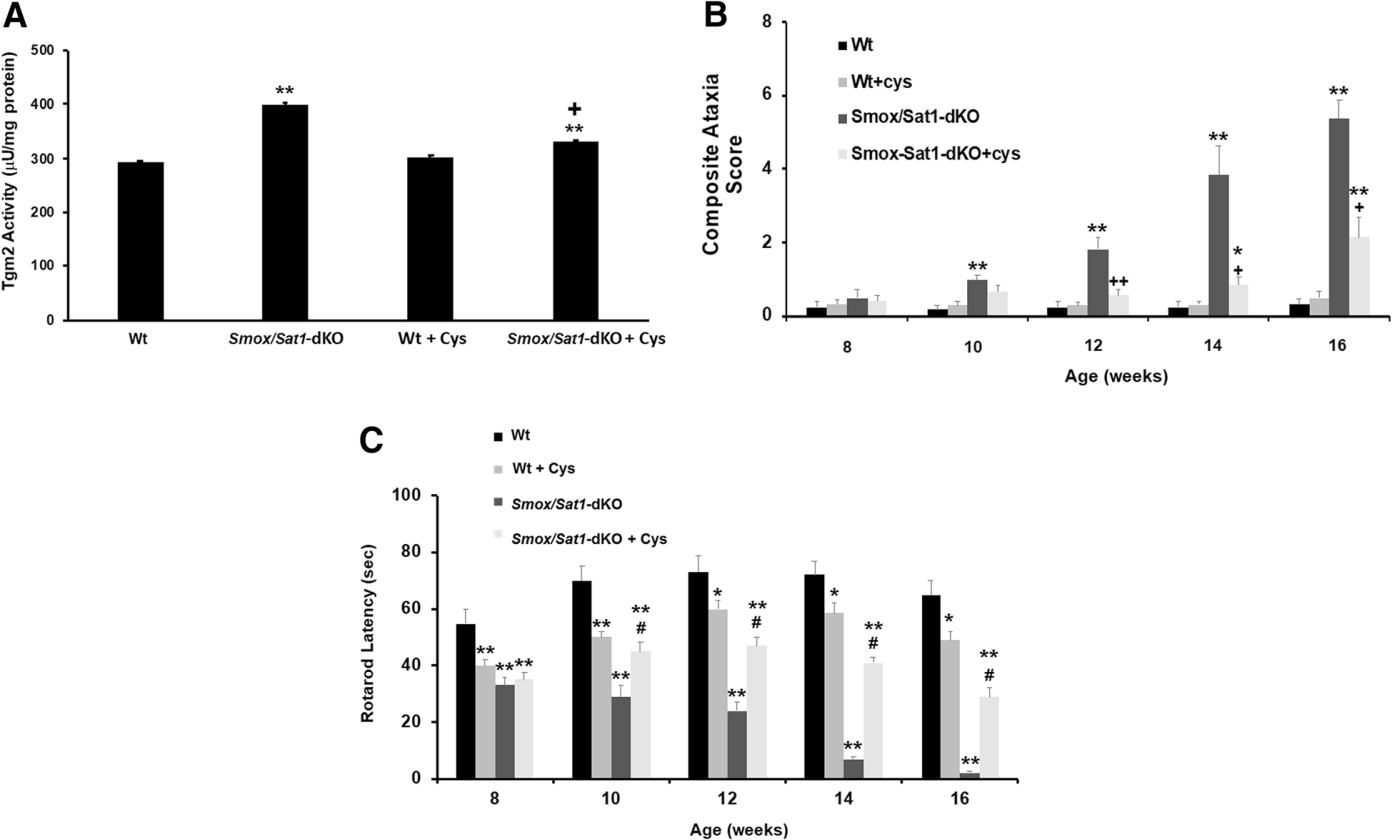
Increased TGM2 activity contributes to the onset and severity of ataxia in *Smox*/*Sat1*-dKO mice. **a** Activity of TGM2 was compared in Wt, Wt + Cys, *Smox*/*Sat1*-dKO and *Smox*/*Sat1*-dKO + Cys mice (*n* = 3/genotype, samples were assayed in triplicate, *t* = 3/sample). The TGM2 activity was significantly higher in both *Smox*/*Sat1*-dKO and *Smox*/*Sat1*-dKO + Cys compared to Wt mice (*p* = 8.15 × 10^−6^ and *p* = 0.00017 respectively). The Cys-treated *Smox*/*Sat1*-dKO mice also had significantly lower cerebellar TGM2 activity compared to untreated *Smox*/*Sat1*-dKO mice (*p* = 0.000044). Each unit of TGM2 activity is defined as “the amount of TGM2 that catalyzes the formation of μmole of hydroxyamate per minute from z-Gln-Gly-OH and hydroxylamine at pH 6.0 at 37 °C.” **b** Comparison of CAS of Wt, Wt + Cys, *Smox*/*Sat1*-dKO, and *Smox*/*Sat1*-dKO + Cys (*n* = 6/genotype). The CAS of Cys-treated *Smox*/*Sat1*-dKO mice in weeks 12 (*p* = 0.0029), 14 (*p* = 0.0304), and 16 (*p* = 0.0262) were significantly improved compared to untreated *Smox*/*Sat1*-dKO mice. **c** Comparison of rotarod latency of fall in Wt, Wt + Cys, *Smox*/*Sat1*-dKO, and *Smox*/*Sat1*-dKO + Cys mice (*n* = 6/genotype). The inhibition of TGM activity significantly improved the rotorod performance of *Smox*/*Sat1*-dKO mice in weeks 10 (*p* = 0.0119), 12 (*p* = 0.000022), 14 (*p* = 0.0015), and 16 (*p* = 0.002) compared to age-matched untreated *Smox*/*Sat1*-dKO mice. (*) Denotes *p* < 0.05, (**) denotes *p* < 0.01 for comparisons between Wt, Wt + Cys, *Smox*/*Sat1*-dKO, and Wt. (+) Denotes *p* < 0.05 and (++) denotes *p* < 0.01 for comparison between Wt and *Smox*/*Sat1*-dKO + Cys mice. (#) Denotes *p* < 0.05 for comparisons between *Smox*/*Sat1*-dKO and *Smox*/*Sat1*-dKO + Cys mice

**Fig. 11:**
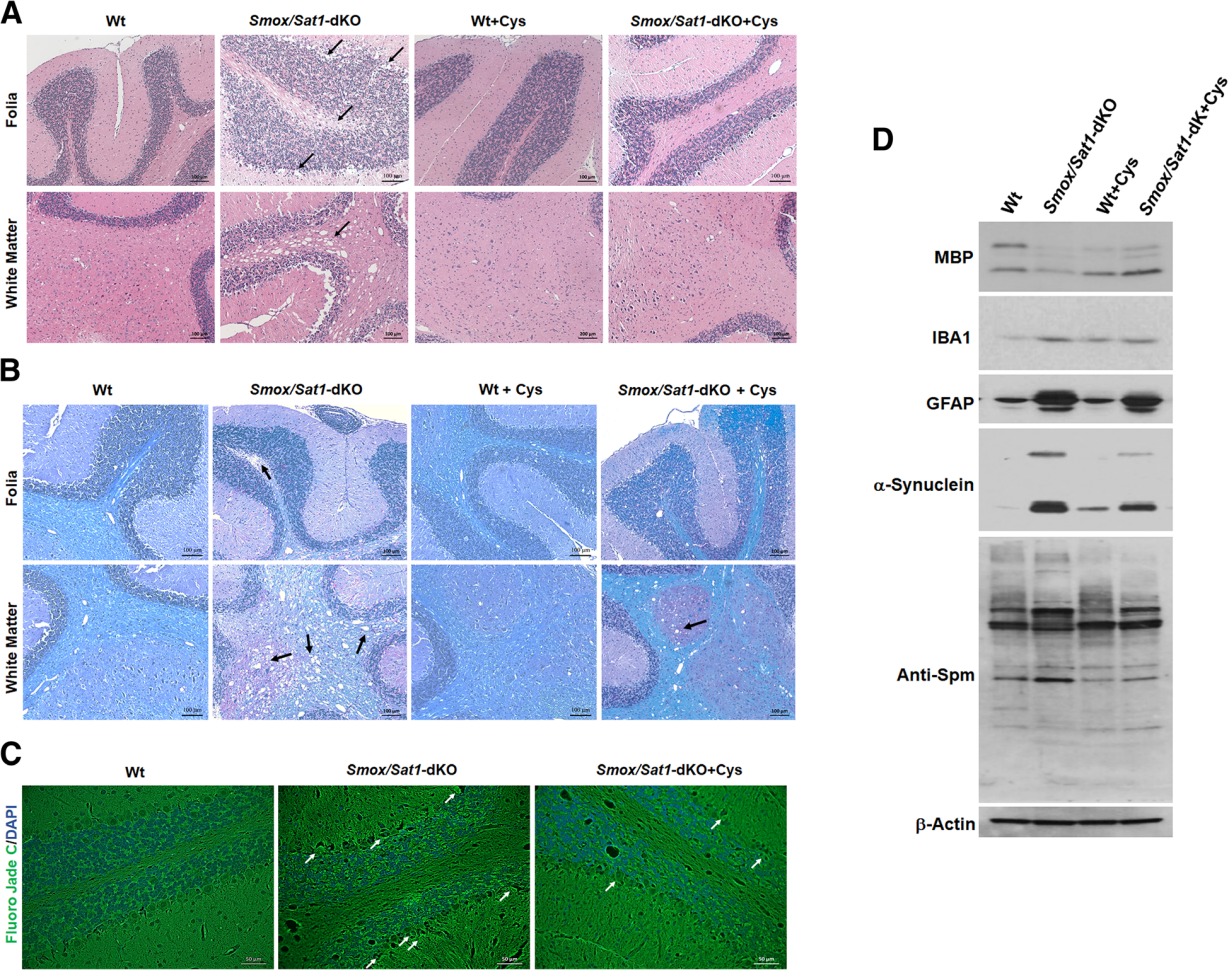
TGM inhibition reduces the cerebellar in *Smox/Sat1*-dKO mice. **a**, **b** Microscopic examination of cerebellar histopathology and demyelination in comparable sections obtained from Wt, Wt + Cys, *Smox/Sat1*-dKO, and *Smox/Sat1*-dKO + Cys mice at 16 weeks of age. Black arrows in A point out the areas of Purkinje cell damage and white matter demyelination. Black arrows in B point to the areas of white matter demyelination. **c** FJC staining of Wt, Wt + Cys, *Smox*/*Sat1*-dKO, and *Smox*/*Sat1*-dKO + Cys mice. White arrows point to degenerating Purkinje cells. **d** Western blot analysis of proteins associated with cerebellar injury in Wt, Wt + Cys, *Smox*/*Sat1*-dKO, and *Smox*/*Sat1*-dKO + Cys mice (results are representative of 3 independent experiments). Western blots contained 50 μg/lane of protein

TGM2 activity contributes to the severity of tissue damage in mouse models of amyotrophic lateral sclerosis, Huntington’s disease, multiple sclerosis, and stroke [39–42]. Therefore, we examined the role of TGMs in the mediating the cerebellar injury in *Smox*/*Sat1*-dKO mice. To test this, the TGM inhibitors cystamine and cysteamine (Cys) were added to the drinking water [43, 44]. TGM2 activity was compared in the cerebellar extracts from Wt mice, *Smox*/*Sat1*-dKO mice, Cys-treated Wt mice, and Cys-treated *Smox*/*Sat1*-dKO mice. Our results indicate that TGM2 activity in the cerebellum of Wt, Cys-treated Wt, and Cys-treated *Smox*/*Sat1*-dKO mice were significantly lower than that of untreated *Smox*/*Sat1*-dKO mice (Fig. 10a). Importantly, CAS and rotarod performances of Cys-treated *Smox*/*Sat1*-dKO mice were also significantly improved compared to untreated *Smox*/*Sat1*-dKO mice (Fig. 10b and c). This reduction in the severity of ataxia was associated with reductions in Purkinje cell damage, leptomeningeal leukocyte infiltration (Fig. 11a), white matter demyelination (Fig. 11a, b, d), neurodegeneration (FJC staining Fig. 11c) and gliosis (IBA1 and GFAP expression, Fig. 11d). Finally, α-Synuclein expression and aggregation, and the extent of protein polyamination, were also reduced in Cys-treated *Smox*/*Sat1*-dKO mice (Fig. 11d). We conclude that elevated spermine levels in concert with enhanced α-Synuclein expression and polyamination mediated by TGMs drive cerebellar damage in *Smox*/*Sat1*-dKO mice.

## Discussion

While enhanced polyamine catabolism is an important mediator of a variety of tissue injuries [9, 10, 45–47], its role in normal physiology was heretofore unclear. To investigate this, we generated mice with simultaneous deletions of *Smox* and *Sat1* genes. Although the expression of *Sat1* and *Smox* is ubiquitous and their expression was globally ablated, *Sat1*/*Smox* deficiency led to strikingly selective damage to the cerebellum and progressive ataxia. Indeed, there are no apparent injuries to any other organs or tissues in *Smox*/*Sat1*-dKO mice (Supplemental Fig. 1), despite the fact that these mice manifest profound, fully penetrant ataxia. Further, our analyses of the development of cerebellar injury and ataxia in *Smox*/*Sat1*-dKO mice indicated that this was due to extensive damage to and degeneration of Purkinje cells (reduced calbindin1 expression and increased fluorescence of Purkinje cells in FJC stained sections), vacuolization of the Purkinje cell layer (histopathology), gliosis (increased GFAP and IBA1 staining), and, at later stages, marked demyelination of white matter, severe edema and leptomeningeal leukocyte infiltration.

Several studies have demonstrated that excess polyamine levels are neurotoxic [6, 48], and that direct intracerebral infusion of spermine can provoke an innate immune response [49]. Studies showing increased expression of SAT1 and SMOX in rats subjected to traumatic brain injury [45], as well as those using SMOX over-expressing transgenic mice, *JoSSMOrec* and Dach-SMOX, support the maladaptive role of enhanced polyamine catabolism in the mediation of brain injury and the regulation of brain function [50–52]. Using different SMOX over-expressing transgenic mice and various excitotoxicity models, it was demonstrated that elevated SMOX levels and the resultant disturbance of polyamine levels increase the severity of seizures caused by kainic acid and pentylentetrazole [50–52]. Of great interest in these models are the induction of oxidative stress (e.g., increased generation H_2_O_2_ and 3-aminopropanal as a result of spermine oxidation), alterations in spermine to spermidine ratios, increased activity of the cystine/glutamate antiporter (System x_c_^−^), and consequent glutamate excitotoxicity. Unlike the *Smox*/*Sat1*-dKO model described in the current manuscript, the root cause of injury in *JoSSMOrec* and Dach-SMOX mice may lie in generation of oxidative stressors, which in concert with other alterations (e.g., increased activity of cystine/glutamate antiporter, System x_c_^−^) may render the SMOX over-expressing mice more prone to seizures [51, 52]. Derangements in polyamine homeostasis are also associated with neurological maladies [53, 54], where for example, mutations in spermine synthase and the ensuing changes in polyamine levels are the cause of Snyder-Robinson Syndrome [54]. Similarly, a mutation in the *ODC1* gene, that leads to the production of a stable form of the protein, also causes severe changes such as macrosomia, macrocephaly, developmental delay, alopecia, spasticity, hypotonia, cutaneous vascular malformation, delayed visual maturation, and sensorineural hearing loss [55]. Polyamines also play key roles in modulating the activity of glutamate receptors, for example in mouse models of alcohol withdrawal neurotoxicity [1, 5, 6, 36]. Toxicity of polyamines in cultured cerebellar granule neurons has also been demonstrated [48], and studies have shown that spermidine as well as spermidine/spermine balance play important roles in the regulation of macrophage polarization and neurological health, respectively [56–58]. Reduced spermidine levels and deficiencies in the hypusination of eIF5A also provoke macrophage activation [58]. Finally, spermidine also has anti-inflammatory and antioxidant functions, enhances mitochondrial metabolic function and respiration, and improves proteostasis and chaperone activity [57], hence reduced spermidine levels may be maladaptive. Collectively, the above studies reveal the importance of maintaining balance in polyamine metabolism and polyamine levels to preserve the integrity of both brain and cerebellar structure and function. Studies by Jain et. al. 2018, showing that the downregulation of polyamine catabolism leads to airway injury, further support the important role of polyamine catabolism in the maintenance of tissue integrity [59]. Here, our analyses revealed increased spermine and reduced spermidine levels in the cerebrum and cerebellum of *Smox*-KO and *Smox*/*Sat1*-dKO versus Wt mice. The comparable polyamine levels in affected (*Smox*/*Sat1*-dKO) and unaffected (*Smox*-KO) mice, as well as affected (cerebellum) and unaffected (cerebrum) areas of the brain led us to conclude that the alterations in polyamine levels contribute to, but are themselves not sufficient, to provoke cerebellar neurotoxicity in *Smox*/*Sat1*-dKO mice.

Expression and immunohistochemical analyses confirmed deleterious effects of the *Smox*/*Sat1* deficiency on Purkinje cells, and provided insights into the mechanisms of cerebellar neurotoxicity in *Smox*/*Sat1*-dKO mice. Specifically, the ataxia observed in *Smox*/*Sat1*-deficient mice appears to initiate with Purkinje cell damage, as evidenced by the reduced expression of genes associated with the viability and function of these neurons, together with attendant gliosis as evidenced by increases in the expression of genes implicated in neurodegenerative M1 microglia and A1 astrocyte polarization [60, 61]. Further, *Smox*/*Sat1*-dKO Purkinje cells displayed marked increases in the expression of *Tgm2*, which has established roles in neurodegeneration [30, 31]. Collectively, these findings suggest the ataxia observed in *Smox*/*Sat1*-dKO mice is initiated as a result of Purkinje cell damage and gliosis (neurodegenerative M1 microglia and A1 astrocyte activation), which is later exacerbated by white matter demyelination and the onset of a reactive immune/inflammatory response (e.g., CD4^+^ and CD8^+^ T cell leptomeningeal infiltrates). The severe damage to Purkinje cells points to the exquisite sensitivity of these cells to the disruption in polyamine metabolism, and to the ensuing sequelae that lead to their destruction in *Smox*/*Sat1*-dKO mice. This selectivity may be explained in part by the high constitutive expression levels of *Sat1* and *Smox* transcripts in the Purkinje cells of the cerebellum, as indicated by expression analyses of mouse brain in the Allen Brain Atlas (“http://mouse.brain-map.org/experiment/show/67881784" and "https://mouse.brain-map.org/gene/show/19992,” Supplemental Fig. 7) The deficiency of SMOX and SAT1 in cells that are the major site of their expression in the cerebellum, in concert with increased α-Synuclein and TGM2 expression, as well as enhanced spermine accumulation, suggests that these alterations act in concert to provoke Purkinje cell damage and ataxia in *Smox*/S*at1*-dKO mice.

The expression and activity of TGMs is elevated in the central nervous system and cerebral spinal fluid of Huntington’s and Parkinson’s disease patients, as well as in the cells of Snyder-Robinson syndrome patients [54, 62, 63]. TGM1 expression is closely associated with tauopathies and corpora amylacea formation in damaged, degenerating neurons [27–29]. Although the expression of *Tgm1* mRNA increases in the cerebellum of *Smox*/*Sat1*-dKO mice, the transcript content is lower than that of *Tgm2* (Fig. 7d). The significantly reduced expression levels of *Tgm1* compared to *Tgm2* mRNA in the cerebellum and cerebrum of *Smox*/*Sat1*-dKO mice suggest that TGM2 plays a more dominant role in the cerebellar injury in these animals. TGM2 has maladaptive roles in neurodegenerative diseases such as Parkinson’s disease, multiple sclerosis (MS), amyotrophic lateral sclerosis, and Huntington’s disease [40–42, 62, 63], and mediates the polyamination of proteins associated with neurodegenerative diseases (e.g., Tauopathies and Huntington's disease) [32, 64, 65]. Further, TGM-mediated polyamination of Phospholipase A2, a mediator of neuroinflammation, increases its activity by 3-fold [66], and its polyamination of tubulin regulates neuronal plasticity [7]. Finally, the ablation of the *Tgm2* gene or inhibition of TGM activity reduces the extent of tissue damage in stroke, MS, and Huntington’s disease [39–41]. Our findings support a model where the elevated levels of spermine and increased polyamination of target proteins such as α-Synuclein by TGM2 in *Smox*/*Sat1*-dKO Purkinje cells drive the development and progression of ataxia.

α-Synuclein is a pre-synaptic protein involved in the regulation of exocytotic vesicles [33, 67], and its expression increases in response to brain injury [68]. Further, α-synuclein oligomerization is important in the pathogenesis of synucleinopathies [69, 70], where it forms neurotoxic oligomers [70–73] that accumulate in and damage Purkinje cells [74–76]. When released into the extracellular space, α-Synuclein oligomers are potent inducers of neuroinflammatory M1 microglia and A1 astrocyte polarization [77, 78]. The expression and aggregation of α-Synuclein increases in the cerebellum of *Smox*/*Sat1*-dKO mice and correlates with the onset of microgliosis and astrocytosis (Fig. 7c). TGMs, in particular TGM2, are known to facilitate the crosslinking and oligomerization of α-Synuclein [63] and polyamines to enhance the oligomerization of α-Synuclein [36–38]. Since inhibition of TGMs in *Smox*/*Sat1*-dKO mice prevents cerebellar injury and ataxia, our findings suggest that an aberrant spermine-TGM2-α-Synuclein-neuroinflammatory circuit is sufficient to provoke Purkinje cell damage, gliosis, and white matter demyelination in *Smox*/*Sat1*-dKO mice, and that this circuit may play an important role in other forms of neurodegeneration.

Purkinje cell damage and gliosis in our model precedes the cerebellar white matter demyelination and the induction of leptomeningeal lymphocyte infiltration that is only observed in severely ataxic animals. This sequence of events lends support to the “inside-out” model of pathogenesis in certain neurodegenerative diseases, including the primary progressive form of MS and leukodystrophies, in which the inflammatory/immune response is a reaction to the initial injury that further aggravates the extent of tissue damage [79].

These studies examine the changes and factors that lead to severe cerebellar injury in *Smox*/*Sat1*-dKO mice; however, the initial alterations that drive the observed neurodegenerative changes need to be elucidated. The combined inactivation of *Smox* and *Sat1* genes and the attendant derangements in polyamine levels and their catabolic pathways can cause structural and metabolic changes (e.g., DNA and histone modifications, protein methylation, and fat metabolism) in *Smox*/*Sat1*-dKO mice that may be responsible for the onset of tissue injury. Deactivation of *Sat1* for example leads to elevated acetyl coenzyme A (acetyl-CoA) levels [13], which can alter fatty acid and steroid synthesis as well as affect protein acetylation (e.g., histones) and gene regulation. Elevated spermine accompanied by a significant decrease in spermidine levels may indicate increased decarboxy *S*-adenosyl methionine (dcAdoMet) usage in *Smox*-KO and *Smox*/*Sat1*-dKO mice. The increased need for dcAdoMet may create a shortfall in the tissue content of its precursor, *S*-adenosyl methionine (AdoMet). The potential reduction in AdoMet levels can alter the methylation of DNA and proteins including histones. The latter changes were proven to be critical in gene regulation and in the pathogenesis of many neurodegenerative diseases [80–82]. Whether or not the spermine synthesis can lead to significant enough changes in dcAdoMet utilization and AdoMet levels as to differentially affect the methylation reactions in *Smox*/*Sat1*-dKO vs. *Smox*-KO mice in is not clear and needs to be examined. Polyamines are also important determinants of DNA and chromatin structure [83–85]. Elevated free spermine levels, as seen in 14+-week-old *Smox*/*Sat1*-dKO mice may contribute to the activation of peptidylargenine deaminases and lead to histone citrullination, which reduces the positive charge of histones, modifies their structure, and alters their interactions with DNA thereby affecting gene regulation [86]. Similarly, significantly higher spermine levels or increased polyamine nuclear aggregate formation, which can arise from increases in putrescine and spermine, can lead to changes in DNA conformation from B to Z [83–85] and profoundly affect gene expression [87]. The simultaneous and combined effects of the alterations listed above may be the initiating factors for the development of severe cerebellar injury in *Smox*/*Sat1*-dKO mice. The individual and concerted role of the aforementioned derangements in the induction of neurodegenerative changes in *Smox*/*Sat1*-dKO is important to our understanding the physiological significance of polyamine catabolic pathways and needs to be elucidated.

*Smox*/*Sat1*-dKO mice develop an age-dependent, progressive ataxia and corresponding extensive cerebellar neurodegeneration, with no evidence of damage to other components of the central nervous system or organs such as kidney, liver, and lung. However, the relatively short life span of the *Smox*/*Sat1*-deficient mice precludes a long-term examination of the potential adverse effects of deficits in polyamine catabolism on the function and structure of the cerebrum and other organs. Accordingly, studies of models with tissue-specific disruption of polyamine catabolism are clearly warranted to better understand its role in maintaining the structural and functional integrity of other organs.

## Conclusions

Based on our results, we propose that prolonged deficits in polyamine catabolism, increased TGM2 activity, and enhanced α-Synuclein expression, polyamination, and aggregation result in the activation of a circuit that leads to cerebellar injury and ataxia in *Smox*/*Sat1*-dKO mice. The data presented here suggest the presence of a close interplay between polyamine catabolism, polyamine levels and TGM-mediated protein polyamination in neurodegenerative conditions.

## Supplementary information

**Additional file 1.**

## Data Availability

The datasets used and/or analyzed in the current study are included in this published article and its supplementary information files. these material are also available from the corresponding author upon request.

## Abbreviations

SAT1: Spermidine/spermine N^1^-acetyltransferase
SMOX: Spermine oxidase
TGM: Transglutaminase
Kir: Inward rectifying potassium channels
ODC: Ornithine decarboxylase
PAOX: N^1^-acetylpolyamine oxidase
Wt: Wild-type
*Smox-*KO: *Smox* knockout
*Sat1-*KO: *Sat1* knockout
*Smox/Sat1-*dKO: *Smox/Sat1* double knockout
CAS: Composite Ataxia Score
DEG: Differentially expressed genes
IPA: Ingenuity pathway analysis
FJC: Fluoro-Jade C
TEM: Transmission electron microscopy
MRI: Magnetic resonance imaging and spectroscopy
H&E: Hematoxylin and eosin
SD: Standard deviation
CALB1: Calbindin D28k
CA8: Carbonic anhydrase 8
PCP2: Purkinje cell protein 2
SLC1A3: Solute carrier family 1 member A3
TRPC3: Transient receptor potential cation channel subfamily C member 3
C1q: Complement factor 1q
Serpine1: Serine peptidase inhibitor clade E member 1
PTX3: Pentraxin 3
C3: Complement factor 3
C4: Complement factor 4
Serpina3n: Serine peptidase inhibitor clade A member 3N
GFAP: Glial fibrillary acidic protein
IBA1: Ionized calcium-binding adapter molecule 1
LFB: Luxol Fast Blue
MBP: Myelin basic protein
Cys: Treatment with cystamine plus cysteamine
AdoMet: *S*-adenosylmethionine
dcAdoMet: Decarboxy *S*-adenosylmethionine
AdoMetDC: *S*-adenosylmethionine decarboxylase

## References

[1] Bowie D (2018). Polyamine-mediated channel block of ionotropic glutamate receptors and its regulation by auxiliary proteins. J Biol Chem.

[2] Lightfoot HL, Hall J (2014). Endogenous polyamine function—the RNA perspective. Nucleic Acids Res.

[3] Pasini A, Caldarera CM, Giordano E (2014). Chromatin remodeling by polyamines and polyamine analogs. Amino Acids.

[4] Masuko T (2003). Polyamine transport, accumulation, and release in brain. J Neurochem.

[5] Caballero R (2010). Flecainide increases Kir2.1 currents by interacting with cysteine 311, decreasing the polyamine-induced rectification. Proc Natl Acad Sci U S A.

[6] Gibson DA (2003). Polyamines contribute to ethanol withdrawal-induced neurotoxicity in rat hippocampal slice cultures through interactions with the NMDA receptor. Alcohol Clin Exp Res.

[7] Song Y (2013). Transglutaminase and polyamination of tubulin: posttranslational modification for stabilizing axonal microtubules. Neuron.

[8] Casero RA, Pegg AE (2009). Polyamine catabolism and disease. Biochem J.

[9] Ivanova S (1998). Cerebral ischemia enhances polyamine oxidation: identification of enzymatically formed 3-aminopropanal as an endogenous mediator of neuronal and glial cell death. J Exp Med.

[10] Zahedi K (2009). Spermidine/spermine-N1-acetyltransferase ablation protects against liver and kidney ischemia-reperfusion injury in mice. Am J Physiol Gastrointest Liver Physiol.

[11] Chaturvedi R (2015). Increased Helicobacter pylori-associated gastric cancer risk in the Andean region of Colombia is mediated by spermine oxidase. Oncogene.

[12] Goodwin AC (2011). Polyamine catabolism contributes to enterotoxigenic Bacteroides fragilis-induced colon tumorigenesis. Proc Natl Acad Sci U S A.

[13] Jell J (2007). Genetically altered expression of spermidine/spermine N1-acetyltransferase affects fat metabolism in mice via acetyl-CoA. J Biol Chem.

[14] Zahedi K (2017). Activation of endoplasmic reticulum stress response by enhanced polyamine catabolism is important in the mediation of cisplatin-induced acute kidney injury. PLoS One.

[15] Guyenet SJ, et al. A simple composite phenotype scoring system for evaluating mouse models of cerebellar ataxia. J Vis Exp. 2010;39. 10.3791/1787 PMC3121238 20495529

[16] Monville C, Torres EM, Dunnett SB (2006). Comparison of incremental and accelerating protocols of the rotarod test for the assessment of motor deficits in the 6-OHDA model. J Neurosci Methods.

[17] Rahmati N (2013). Slc26a11 is prominently expressed in the brain and functions as a chloride channel: expression in Purkinje cells and stimulation of V H(+)-ATPase. Pflugers Arch.

[18] Rahmati N, et al. SLC26A11 (KBAT) in Purkinje cells is critical for inhibitory transmission and contributes to locomotor coordination. eNeuro. 2016;3(3). 10.1523/ENEURO.0028-16.2016 PMC4908300 27390771

[19] Schmued LC (2005). Fluoro-Jade C results in ultra high resolution and contrast labeling of degenerating neurons. Brain Res.

[20] Rabenstein M (2018). Impact of reduced cerebellar EAAT expression on Purkinje cell firing pattern of NPC1-deficient mice. Sci Rep.

[21] Perkins EM, et al. Loss of cerebellar glutamate transporters EAAT4 and GLAST differentially affects the spontaneous firing pattern and survival of Purkinje cells. Hum Mol Genet. 2018. 10.1093/hmg/ddy169 PMC6049029 29741614

[22] Hartmann J, Konnerth A (2015). TRPC3-dependent synaptic transmission in central mammalian neurons. J Mol Med (Berl).

[23] Wu B, et al. TRPC3 is a major contributor to functional heterogeneity of cerebellar Purkinje cells. Elife. 2019;8. 10.7554/eLife.45590 PMC6733575 31486767

[24] Andringa G (2004). Tissue transglutaminase catalyzes the formation of alpha-synuclein crosslinks in Parkinson's disease. FASEB J.

[25] Dudek SM, Johnson GV (1994). Transglutaminase facilitates the formation of polymers of the beta-amyloid peptide. Brain Res.

[26] Miller ML, Johnson GV (1995). Transglutaminase cross-linking of the tau protein. J Neurochem.

[27] Tripathy D (2020). Increased transcription of transglutaminase 1 mediates neuronal death in in vitro models of neuronal stress and Abeta1-42-mediated toxicity. Neurobiol Dis.

[28] Wilhelmus MM (2012). Transglutaminase 1 and its regulator tazarotene-induced gene 3 localize to neuronal tau inclusions in tauopathies. J Pathol.

[29] Wilhelmus MM (2011). Novel role of transglutaminase 1 in corpora amylacea formation?. Neurobiol Aging.

[30] Ientile R, Curro M, Caccamo D (2015). Transglutaminase 2 and neuroinflammation. Amino Acids.

[31] Jeitner TM (2009). Transglutaminases and neurodegeneration. J Neurochem.

[32] Andre W (2017). Identification of brain substrates of transglutaminase by functional proteomics supports its role in neurodegenerative diseases. Neurobiol Dis.

[33] Cabin DE (2002). Synaptic vesicle depletion correlates with attenuated synaptic responses to prolonged repetitive stimulation in mice lacking alpha-synuclein. J Neurosci.

[34] Hong L (1998). The cDNA cloning and ontogeny of mouse alpha-synuclein. Neuroreport.

[35] Sugimura Y (2006). Screening for the preferred substrate sequence of transglutaminase using a phage-displayed peptide library: identification of peptide substrates for TGASE 2 and Factor XIIIA. J Biol Chem.

[36] Antony T (2003). Cellular polyamines promote the aggregation of alpha-synuclein. J Biol Chem.

[37] Fernandez CO (2004). NMR of alpha-synuclein-polyamine complexes elucidates the mechanism and kinetics of induced aggregation. EMBO J.

[38] Lewandowski NM (2010). Polyamine pathway contributes to the pathogenesis of Parkinson disease. Proc Natl Acad Sci U S A.

[39] Colak G, Johnson GV (2012). Complete transglutaminase 2 ablation results in reduced stroke volumes and astrocytes that exhibit increased survival in response to ischemia. Neurobiol Dis.

[40] McConoughey SJ (2010). Inhibition of transglutaminase 2 mitigates transcriptional dysregulation in models of Huntington disease. EMBO Mol Med.

[41] Oono M (2014). Transglutaminase 2 accelerates neuroinflammation in amyotrophic lateral sclerosis through interaction with misfolded superoxide dismutase 1. J Neurochem.

[42] van Strien ME (2015). Tissue transglutaminase contributes to experimental multiple sclerosis pathogenesis and clinical outcome by promoting macrophage migration. Brain Behav Immun.

[43] Jeitner TM, Pinto JT, Cooper AJL. Cystamine and cysteamine as inhibitors of transglutaminase activity in vivo. Biosci Rep. 2018;(5):38. 10.1042/BSR20180691 PMC6123069 30054429

[44] Pinto JT (2005). Treatment of YAC128 mice and their wild-type littermates with cystamine does not lead to its accumulation in plasma or brain: implications for the treatment of Huntington disease. J Neurochem.

[45] Zahedi K (2010). Polyamine catabolism is enhanced after traumatic brain injury. J Neurotrauma.

[46] Zahedi K (2003). Expression of SSAT, a novel biomarker of tubular cell damage, increases in kidney ischemia-reperfusion injury. Am J Physiol Ren Physiol.

[47] Dogan A (1999). Effects of MDL 72527, a specific inhibitor of polyamine oxidase, on brain edema, ischemic injury volume, and tissue polyamine levels in rats after temporary middle cerebral artery occlusion. J Neurochem.

[48] Segal JA, Skolnick P (2000). Spermine-induced toxicity in cerebellar granule neurons is independent of its actions at NMDA receptors. J Neurochem.

[49] Soulet D, Rivest S (2003). Polyamines play a critical role in the control of the innate immune response in the mouse central nervous system. J Cell Biol.

[50] Cervelli M (2013). A New Transgenic mouse model for studying the neurotoxicity of spermine oxidase dosage in the response to excitotoxic injury. PLoS One.

[51] Leonetti A (2020). Epileptic seizures and oxidative stress in a mouse model over-expressing spermine oxidase. Amino Acids.

[52] Pietropaoli S (2018). Glutamate excitotoxicity linked to spermine oxidase overexpression. Mol Neurobiol.

[53] Minois N, Carmona-Gutierrez D, Madeo F (2011). Polyamines in aging and disease. Aging (Albany NY).

[54] Murray-Stewart T, et al. Polyamine homeostasis in Snyder-Robinson syndrome. Med Sci (Basel). 2018;6(4). 10.3390/medsci6040112 PMC6318755 30544565

[55] Bupp CP (2018). Novel de novo pathogenic variant in the ODC1 gene in a girl with developmental delay, alopecia, and dysmorphic features. Am J Med Genet A.

[56] Gupta VK (2016). Spermidine suppresses age-associated memory impairment by preventing Adverse increase of presynaptic active zone size and release. PLoS Biol.

[57] Madeo F, et al. Spermidine in health and disease. Science. 2018;359(6374). 10.1126/science.aan2788 29371440

[58] Puleston DJ (2019). Polyamines and eIF5A Hypusination modulate mitochondrial respiration and macrophage activation. Cell Metab.

[59] Jain V (2018). Reduction in polyamine catabolism leads to spermine-mediated airway epithelial injury and induces asthma features. Allergy.

[60] Franco R, Fernandez-Suarez D (2015). Alternatively activated microglia and macrophages in the central nervous system. Prog Neurobiol.

[61] Liddelow SA, Barres BA (2017). Reactive astrocytes: production, function, and therapeutic potential. Immunity.

[62] Jeitner TM (2008). Increased levels of gamma-glutamylamines in Huntington disease CSF. J Neurochem.

[63] Junn E (2003). Tissue transglutaminase-induced aggregation of alpha-synuclein: Implications for Lewy body formation in Parkinson's disease and dementia with Lewy bodies. Proc Natl Acad Sci U S A.

[64] Lesort M (2002). Does tissue transglutaminase play a role in Huntington's disease?. Neurochem Int.

[65] Tucholski J, Kuret J, Johnson GV (1999). Tau is modified by tissue transglutaminase in situ: possible functional and metabolic effects of polyamination. J Neurochem.

[66] Cordella-Miele E (1993). Transglutaminase-catalyzed incorporation of polyamines into phospholipase A2. J Biochem.

[67] Logan T (2017). alpha-Synuclein promotes dilation of the exocytotic fusion pore. Nat Neurosci.

[68] Kim T (2016). Poststroke Induction of alpha-synuclein mediates ischemic brain damage. J Neurosci.

[69] Galvin JE, Lee VM, Trojanowski JQ (2001). Synucleinopathies: clinical and pathological implications. Arch Neurol.

[70] Wong YC, Krainc D (2017). alpha-synuclein toxicity in neurodegeneration: mechanism and therapeutic strategies. Nat Med.

[71] Roostaee A (2013). Aggregation and neurotoxicity of recombinant alpha-synuclein aggregates initiated by dimerization. Mol Neurodegener.

[72] Spillantini MG, Goedert M (2018). Neurodegeneration and the ordered assembly of alpha-synuclein. Cell Tissue Res.

[73] Luk KC (2012). Intracerebral inoculation of pathological alpha-synuclein initiates a rapidly progressive neurodegenerative alpha-synucleinopathy in mice. J Exp Med.

[74] Mori F (2003). Alpha-synuclein accumulates in Purkinje cells in Lewy body disease but not in multiple system atrophy. J Neuropathol Exp Neurol.

[75] Solmaz V (2017). Accumulation of alpha-Synuclein in cerebellar purkinje cells of diabetic rats and its potential relationship with inflammation and oxidative stress markers. Neurol Res Int.

[76] Sekiya H (2019). Wide distribution of alpha-synuclein oligomers in multiple system atrophy brain detected by proximity ligation. Acta Neuropathol.

[77] Lee HJ, Kim C, Lee SJ (2010). Alpha-synuclein stimulation of astrocytes: Potential role for neuroinflammation and neuroprotection. Oxidative Med Cell Longev.

[78] Zhang W (2005). Aggregated alpha-synuclein activates microglia: a process leading to disease progression in Parkinson’s disease. FASEB J.

[79] Stys PK (2012). Will the real multiple sclerosis please stand up?. Nat Rev Neurosci.

[80] Berson A (2018). Epigenetic Regulation in Neurodegenerative Diseases. Trends Neurosci.

[81] Celarain N, Tomas-Roig J (2020). Aberrant DNA methylation profile exacerbates inflammation and neurodegeneration in multiple sclerosis patients. J Neuroinflammation.

[82] Lu H (2013). DNA methylation, a hand behind neurodegenerative diseases. Front Aging Neurosci.

[83] D'Agostino L, di Pietro M, Di Luccia A (2005). Nuclear aggregates of polyamines are supramolecular structures that play a crucial role in genomic DNA protection and conformation. FEBS J.

[84] Hasan R, Alam MK, Ali R (1995). Polyamine induced Z-conformation of native calf thymus DNA. FEBS Lett.

[85] Iacomino G (2011). DNA is wrapped by the nuclear aggregates of polyamines: the imaging evidence. Biomacromolecules.

[86] Brooks WH (2013). Increased polyamines alter chromatin and stabilize autoantigens in autoimmune diseases. Front Immunol.

[87] Oh DB, Kim YG, Rich A (2002). Z-DNA-binding proteins can act as potent effectors of gene expression in vivo. Proc Natl Acad Sci U S A.

