# Supplementary material for: Ablation of polyamine catabolic enzymes provokes Purkinje cell damage, neuroinflammation, and severe ataxia
Source: J Neuroinflammation. 2020 Oct 14;17:301. doi: 10.1186/s12974-020-01955-6 (PMC7559641; doi:10.1186/s12974-020-01955-6)
Supplement: Supplementary file 3 — Additional file 1. [file 12974_2020_1955_MOESM1_ESM.zip › SUPPLEMENTAL DATA.docx]

**SUPPLEMENTAL DATA**

**Ablation of Polyamine Catabolic Enzymes Provokes Purkinje Cell Damage, Neuroinflammation and Severe Ataxia**

Kamyar Zahedi^1, 2, 3*+^, Marybeth Brooks^2+^, Sharon Barone^1, 2, 3+^, Negah Rahmati^4^, Tracy Murray Stewart^5^, Matthew Dunworth^5^, Christina Destefano-Shields^5^, Nupur Dasgupta^6^, Steve Davidson^7^, Diana M. Lindquist^8^, Christine E. Fuller^9^, Roger D. Smith^10^, John L. Cleveland^11, 12^, Robert A. Casero Jr.^5^, and Manoocher Soleimani^1, 2, 3*+^.

Kamyar Zahedi, Ph.D.

Department of Internal Medicine, Division of Nephrology

University of New Mexico College of Medicine

915 Camino de Salud, Bldg 289, IDTC 3315

Albuquerque, NM 87113

[KZahedi@salud.unm.edu](mailto:Soleimm@ucmail.uc.edu)

ORCID: 0000-0002-4257-5107

or

Manoocher Soleimani, M.D.

Department of Internal Medicine, Division of Nephrology

University of New Mexico College of Medicine

915 Camino de Salud, Bldg 289, IDTC 3315

Albuquerque, NM 87113

[MSoleimani@salud.unm.edu](mailto:Soleimm@ucmail.uc.edu)

ORCID: [0000-0003-4909-4469](https://orcid.org/0000-0003-4909-4469)

**The Supplementl data includes:**

Supplement Table 1

Supplemental Figures 1-2.

Captions for supplemental data sets1, 2 and 3. Supplemental datasets are provided as separate EXEL files.

Supplemental Figures 3-7

**Supplemental Table 1. List and source of antibodies.**


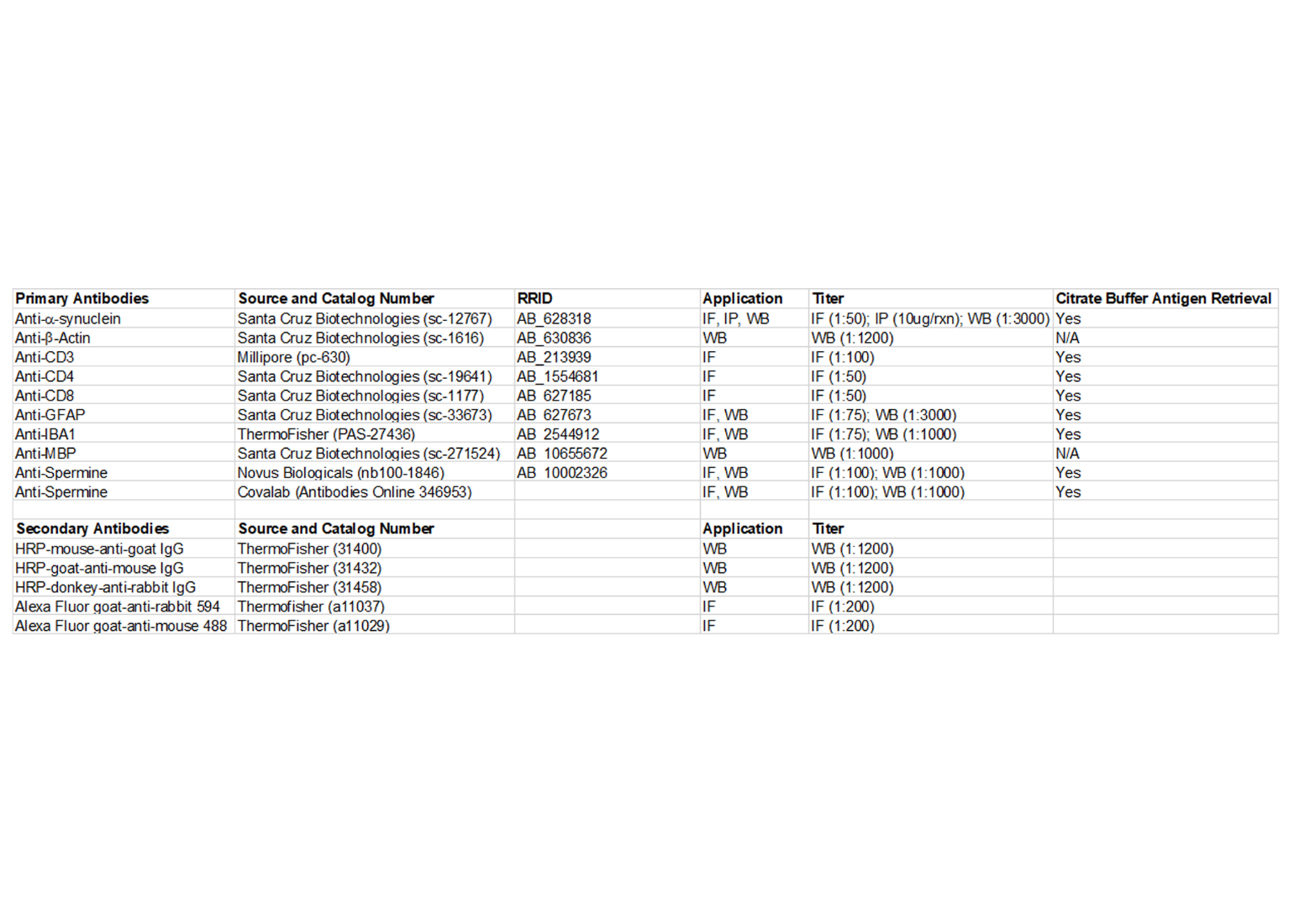


**
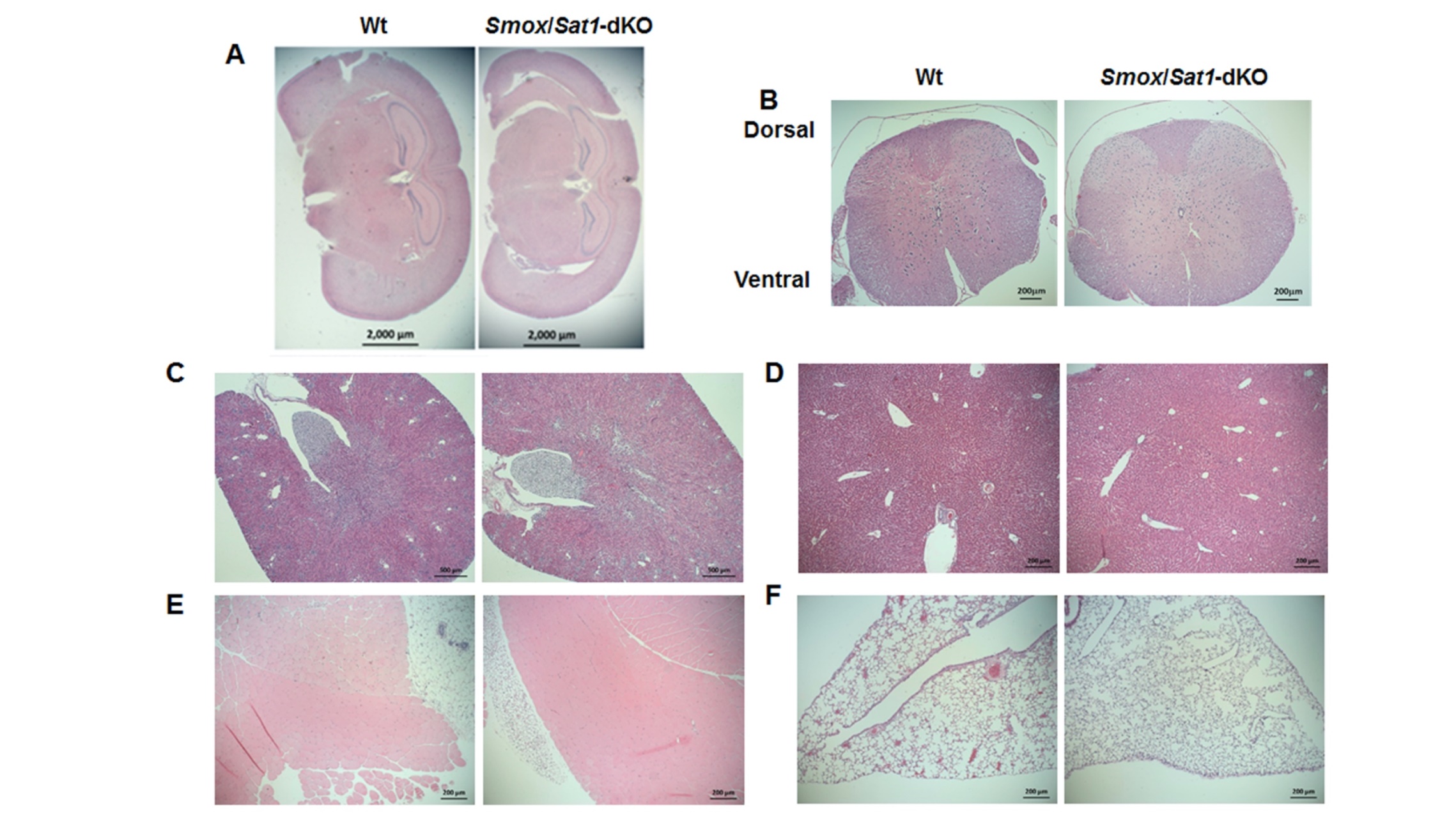

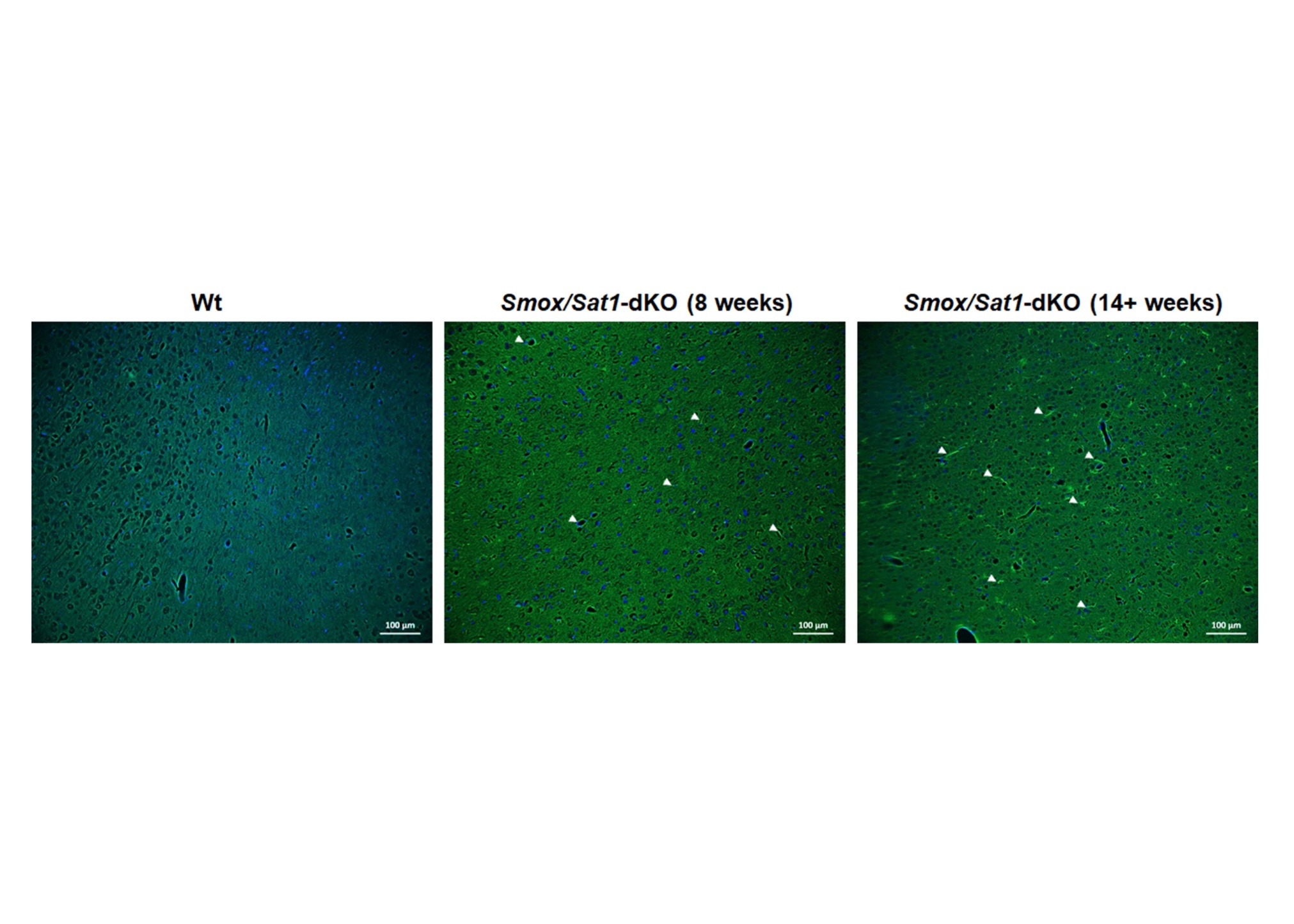
**

**Supplemental Figure 1. Histological analyses of tissues of Wt and *Smox/Sat1*-dKO mice.** Histological examination of A) cerebrum, B) spinal cord, C) kidney, D) liver, E) muscle and F) lung of 14+ weeks old Wt (*left panels*) and *Smox/Sat1*-dKO (*right panels*) mice did not reveal any discernable morphological differences.

**Supplemental Figure 2. FJC staining in the cerebrum of *Smox/Sat1*-dKO mice.** FJC staining reveals the presence of neuronal damage in the cerebrum of 8 and 14 weeks old mice (arrowheads).

**Data set S1. List of differentially expressed genes in the cerebellum of Wt vs. ataxic-*Smox/Sat1*-dKO mice.**

**Data set S2. List of pathways associated with differentially expressed genes identified by RNA-Seq analysis.**

**Data set S3. List of disease and functions associated with differentially expressed genes identified by RNA-Seq analysis.**

**
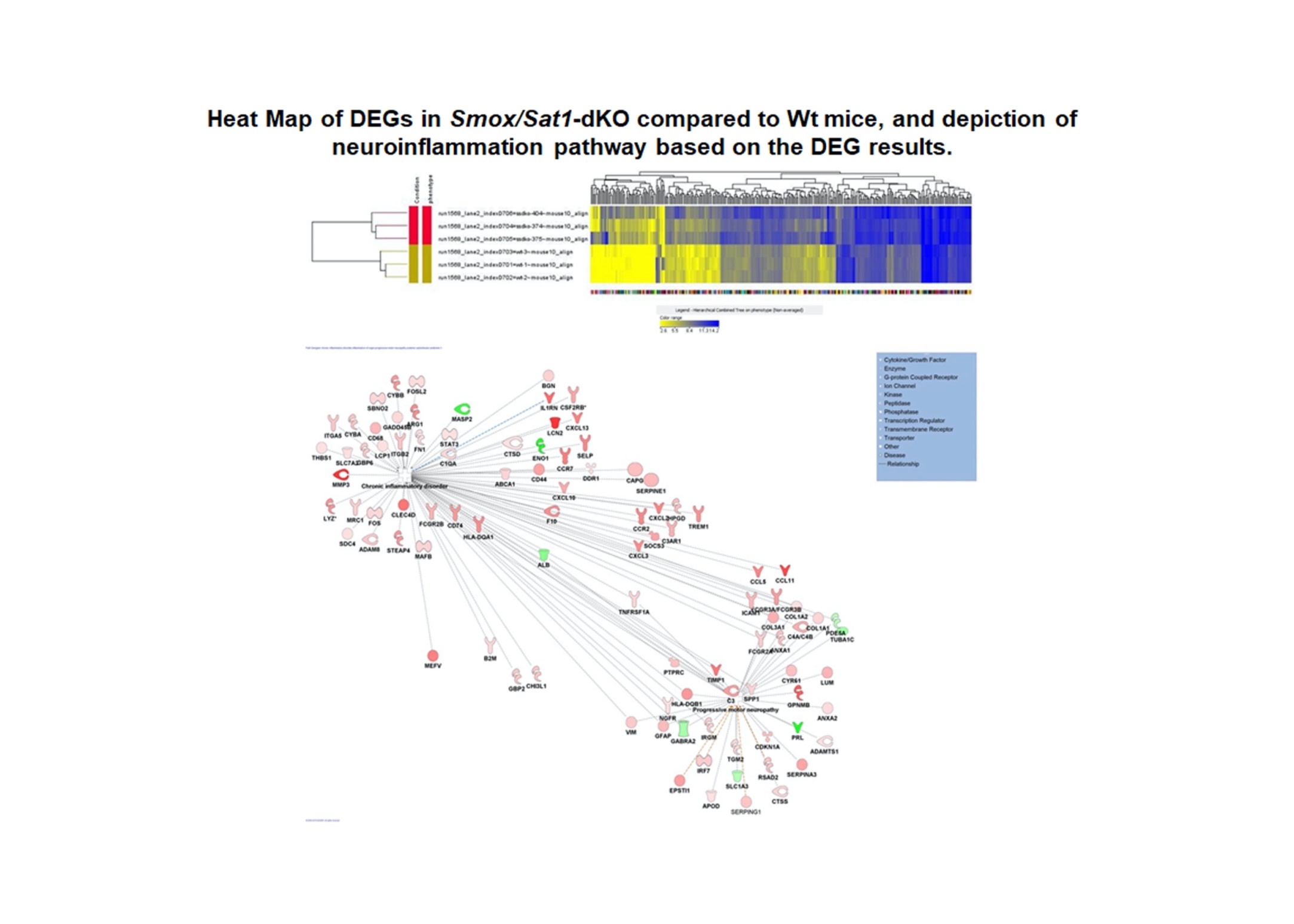
**

**Supplemental Figure 3. RNA-Seq data of cerebellar *Smox/Sat1*-dKO tissue reveals links with chronic inflammation and development of progressive motor deficits.** *Top panel*, represents the heat map generated from the results of RNA-Seq analysis. Bottom panel, schematic illustrates the pathways that are activated in the latter stages of ataxia in dKO mice.

**
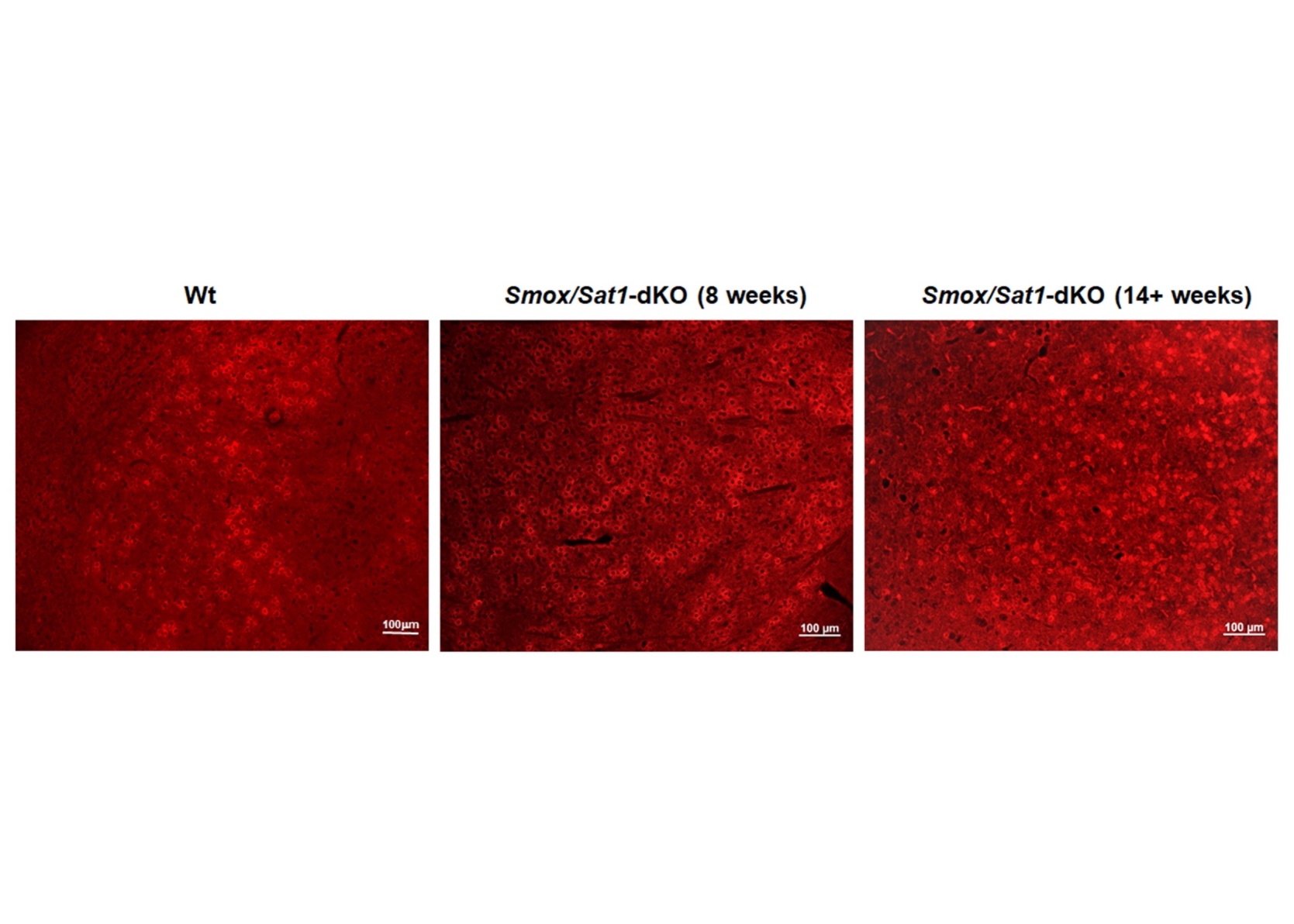
Supplemental Figure 4. Cerebral CALB1 expression in Wt and *Smox/Sat1*-dKO mice.** Expression of CALB1 was not altered in Wt and *Smox/Sat1*-dKO mice with at 8 and 14+ weeks of age.

**
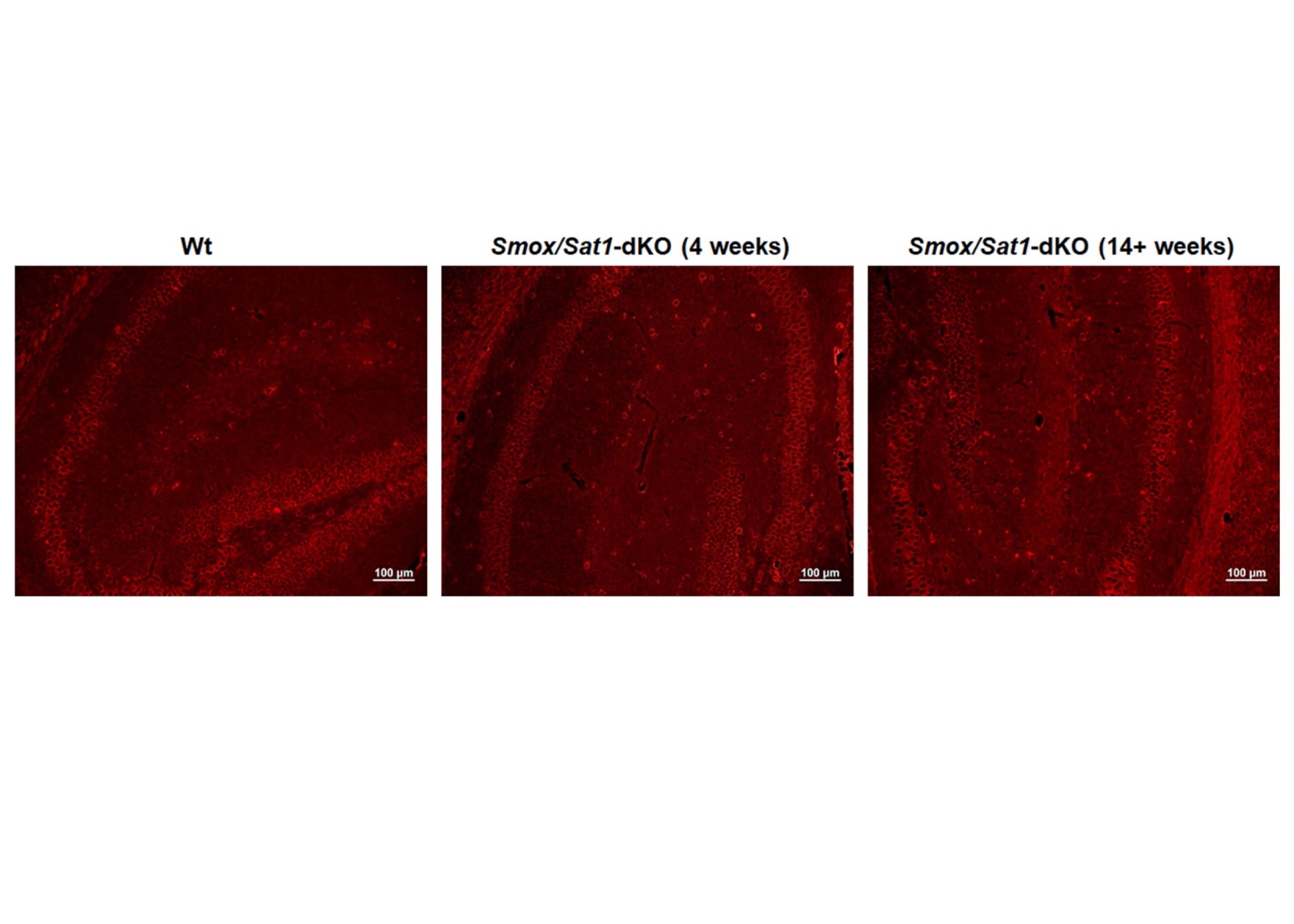
Supplemental Figure 5. TGM2 expression in the cerebrum of Wt and *Smox/Sat1*-dKO mice.** Expression of TGM2 was not altered in the cerebrum of Wt, 4 weeks old and 14+ weeks old *Smox/*Sat1-dKO mice.

**
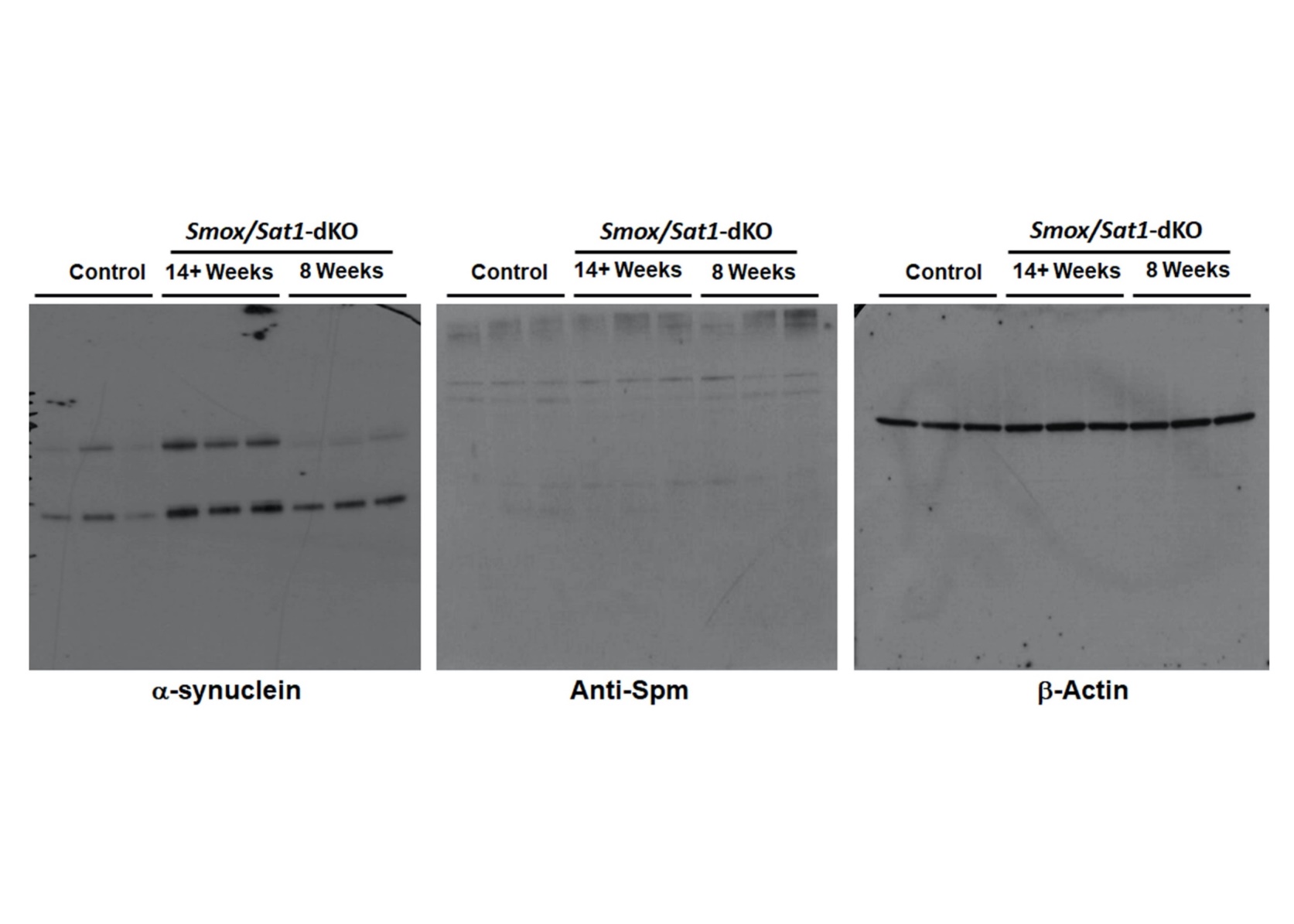
Supplemental Figure 6. α-synuclein expression and protein polyamination in the cerebrum of Wt, 14+ weeks old and 8 weeks old *Smox/Sat1*-dKO mice.** The expression of α-synuclein and alterations in protein polyamination were compared in the protein extracts from the cerebrum of Wt, 8 weeks old and 14+ weeks old *Smox/*Sat1-dKO mice.

**
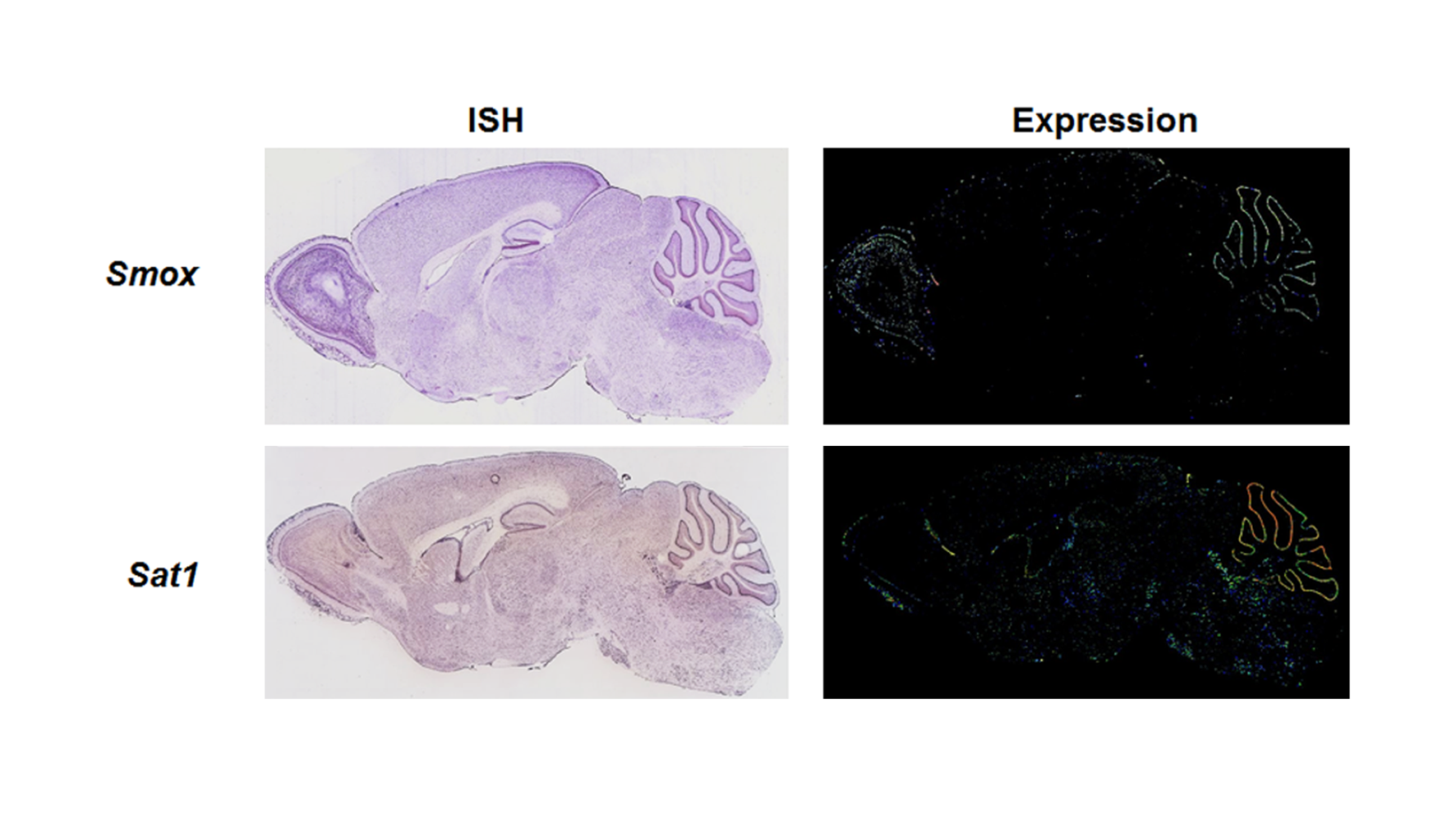
Supplemental Figure 7. *In Situ* hybridization and expression maps of Smox and Sat1 gene in mouse brain.** These photographs obtained from Allen Brain Atlas ("<http://mouse.brain-map.org/experiment/show/67881784>" and "<https://mouse.brain-map.org/gene/show/19992>") depict the expression patterns of *Smox* and *Sat1* transcripts in mouse brain.
